# Plant NLR immune receptor Tm-2^2^ activation requires NB-ARC domain-mediated self-association of CC domain

**DOI:** 10.1371/journal.ppat.1008475

**Published:** 2020-04-27

**Authors:** Junzhu Wang, Tianyuan Chen, Meng Han, Lichao Qian, Jinlin Li, Ming Wu, Ting Han, Jidong Cao, Ugrappa Nagalakshmi, John P. Rathjen, Yiguo Hong, Yule Liu

**Affiliations:** 1 MOE Key Laboratory of Bioinformatics, Center for Plant Biology, Tsinghua-Peking Joint Center for Life Sciences, School of Life Sciences, Tsinghua University, Beijing, China; 2 Department of Plant Biology and The Genome Center, College of Biological Sciences, University of California at Davis, CA, United States of America; 3 Research School of Biology, The Australian National University, Acton, Australia; 4 Research Centre for Plant RNA Signaling, College of Life and Environmental Sciences, Hangzhou Normal University, Hangzhou, China; Agriculture and Agri-Food Canada, CANADA

## Abstract

The nucleotide-binding, leucine-rich repeat-containing (NLR) class of immune receptors of plants and animals recognize pathogen-encoded proteins and trigger host defenses. Although animal NLRs form oligomers upon pathogen recognition to activate downstream signaling, the mechanisms of plant NLR activation remain largely elusive. Tm-2^2^ is a plasma membrane (PM)-localized coiled coil (CC)-type NLR and confers resistance to *Tobacco mosaic virus* (TMV) by recognizing its viral movement protein (MP). In this study, we found that Tm-2^2^ self-associates upon recognition of MP. The CC domain of Tm-2^2^ is the signaling domain and its function requires PM localization and self-association. The nucleotide-binding (NB-ARC) domain is important for Tm-2^2^ self-interaction and regulates activation of the CC domain through its nucleotide-binding and self-association. (d)ATP binding may alter the NB-ARC conformation to release its suppression of Tm-2^2^ CC domain-mediated cell death. Our findings provide the first example of signaling domain for PM-localized NLR and insight into PM-localized NLR activation.

## Author summary

Nucleotide-binding, leucine-rich repeat proteins (NLR) can function as immune receptors of plants and animals and confer resistance against pathogens. However, despite their importance in immunity, the activation mechanism of PM-localized NLRs remains largely elusive. In this study, we demonstrate that CC domain is the signaling domain for inducing cell death for PM-localized NLR Tm-2^2^. Further, we report that nucleotide-binding (NB-ARC) domain is important for Tm-2^2^ self-association and regulates activation of CC domain through its nucleotide-binding and self-association. Our findings provide the first example of signaling domain for PM-localized NLR and insight into PM-localized NLR activation.

## Introduction

Plants use multi-layered sensing systems to defend against pathogens [1]. One such system is based on plant resistance (R) proteins, which directly or indirectly recognize specific pathogen virulence proteins called effectors [2] and activate immune responses that often include localized cell death to restrict the pathogen to the infection site [3].

Most plant R proteins are immune receptors that possess nucleotide-binding (NB) domain and leucine-rich repeat-containing (LRR) domains [4]. They are part of a broad family conserved between plants and animals known as Nod-like receptors (NLR) [5]. The central nucleotide-binding domain functions as a molecular switch that regulates NLR activation by binding and hydrolyzing nucleotides [6, 7], and is also known as NB-ARC domain shared by Apaf-1, CED-4 and most plant R proteins [8]. The NB-ARC domain can be further separated into NB, ARC1 and ARC2 subdomains, and contains several conserved motifs such as the P-loop and the MHD motif [9–12]. The conserved MHD motif in the ARC2 subdomain coordinates nucleotide-binding and also regulates subdomain interactions [10, 13]. When D is changed to V in the MHD motif, the mutant NLR preferentially binds ATP than ADP, resulting in conformational changes and auto-activation [7, 10]. The P-loop motif in the NB subdomain corresponds to a flexible glycine-rich loop containing a highly conserved lysine that contacts the phosphate of ADP or (d)ATP [14–16]. The RNBS-B motif in the NB subdomain also takes part in (d)ATP binding and its arginine senses the presence of the γ–phosphate [6, 14]. The C-terminal LRR domain of NLRs is usually responsible for effector recognition. In addition, the LRR may interact with the NB-ARC domain to inhibit NLR autoactivation [17, 18]. The N-termini of plant NLRs typically contain either a CC (coiled-coil domain) or a TIR (Toll/Interleukin-1 receptor) domain. Overexpression of isolated CC or TIR domains of several NLR proteins is reported to induce cell death [19–25]. NB subdomain of the CC-NLR Rx and NB-ARC domain of the SD-CC-NLR (Solanaceae domain-CC-NLR) Sw-5 can induce cell death [26, 27]. However, to date a domain responsible for induction of cell death and activation of defense signaling of a plasma membrane-localized NLR is largely unclear.

Activated animal NLRs usually oligomerize and form high molecular weight complexes known as inflammasomes and recruit caspases to stimulate immune signaling [28]. Recognition of pathogen-encoded effectors leads to altered intramolecular interactions within the NLR, and the subsequent replacement of ADP by (d)ATP within the NB-ARC domain results in NLR oligomerization; this induced oligomerization model is well established in animal NLR activation and oligomerization is a key step in the activation of animal NLRs [29, 30]. Structural analysis of animal NLRs indicates that the NB-ARC domain is the primary region for NLR self-association [31]. On the other hand, less is known about domains involved in plant NLR oligomerization. Several NLRs have been demonstrated to self-associate upon specific recognition of their corresponding effectors, including TIR-NLRs (tobacco N and Arabidopsis RPP1) and a CC-NLR (Arabidopsis ZAR1) [14, 25, 32]. Some CC-NLRs such as Arabidopsis RPS5, RPM1 and barley MLA1 appear to self-associate in the absence of their cognate effectors [19, 20, 33–37].

In addition, N-terminal domains of several plant NLRs can self-associate and this self-association is essential for their function, supporting the notion that oligomerization is an important step in NLR-mediated immune signaling [35, 38, 39]. TIR domains can form homo- and hetero-dimers [40–42]. Compared to TIR domains, CC domains are more variable in structure. The MLA10 CC domain (amino acids (aa) 5–120) crystallizes as a homodimer adopting a helix–loop–helix fold [19], while NMR studies suggest that the CC domain (aa 6–120) of its orthologous protein Sr33 is a monomeric four-helix bundle similar to the crystal structure of the Rx CC domain [24, 43]. Further biophysical characterization by light scattering assays indicates that the CC regions of MLA10, Rx and Sr33 are monomeric in solution, similar to the crystal or NMR structures of Rx and Sr33, but different from that of MLA10 [24]. However, shorter monomeric CC domains fail to induce cell death compared to longer CC domains that possess a complete C-terminal fourth helix and can dimerize *in vivo* and *in vitro* [24]. Consistent with this, the recently reported ZAR1 structure shows that the CC domain of the inactive ZAR1 monomer forms a four-helix bundle which is similar to that of Rx and Sr33; ZAR1 adopts fold switching during the activation process and the CC domains form an α-helical barrel with their N-termini projecting out of the wheel-defined barrel plane in the active pentamer ZAR1 resistosome [14, 15]. However, the knowledge about how the structure of CC changes during activation and how other domains function in this process is very limited.

The tomato CC-NLR Tm-2^2^ confers extreme resistance against *Tobamoviruses* such as *Tobacco mosaic virus* (TMV) and *Tomato mosaic virus* (ToMV) [44, 45] by recognizing the viral movement protein (MP) which facilitates cell-to-cell movement of the virus through plasmodesmata (PD) [46, 47]. Tm-2^2^ functions at the plasma membrane (PM) and recognizes viral MP independent of its PD localization [48]. Besides Tm-2^2^, several Arabidopsis CC-NLR (RPS5 and RPM1) and rice CC-NLR (Pit) are reported to function at the PM [17, 49, 50]. In addition, Arabidopsis CC-NLR ZAR1’s PM association is enhanced after effector recognition [14] although it is also localized to nucleus and endoplasmic reticulum besides PM [51]. However, the knowledge about PM-localized CC-NLR signaling domain is still very limited. The RPS5 CC domain is sufficient to target fusion proteins to the PM, but only the CC-NB-ARC domain can induce cell death [17, 33]. The RPM1 CC domain also localizes to the PM, but all domains are critical to its function [34]. In addition, the isolated ZAR1 CC domain induces cell death but it localizes to the nucleus and the cytoplasm [23]. Although the CC domains of some cytosolic CC-NLRs appear to trigger cell death, there is no report for PM-localized CC domains.

In this study, we used Tm-2^2^ as a model to investigate how PM-localized NLRs are regulated during activation. We found that Tm-2^2^ self-associates post effector recognition. Although a sole CC domain was able to induce cell death once localized to the PM, CC self-association was essential for its function. Moreover, we found that the wild-type Tm-2^2^ NB-ARC domain inhibited CC-mediated cell death, while an auto-active MHD motif mutation in the NB-ARC domain enhanced CC domain function by helping its self-association. These results suggest that the plant immune receptor Tm-2^2^ requires NB-ARC domain-mediated oligomerization of the CC domain at the PM for its signaling.

## Results

### The Tm-2^2^ NLR self-associates upon activation

**Fig 1:**
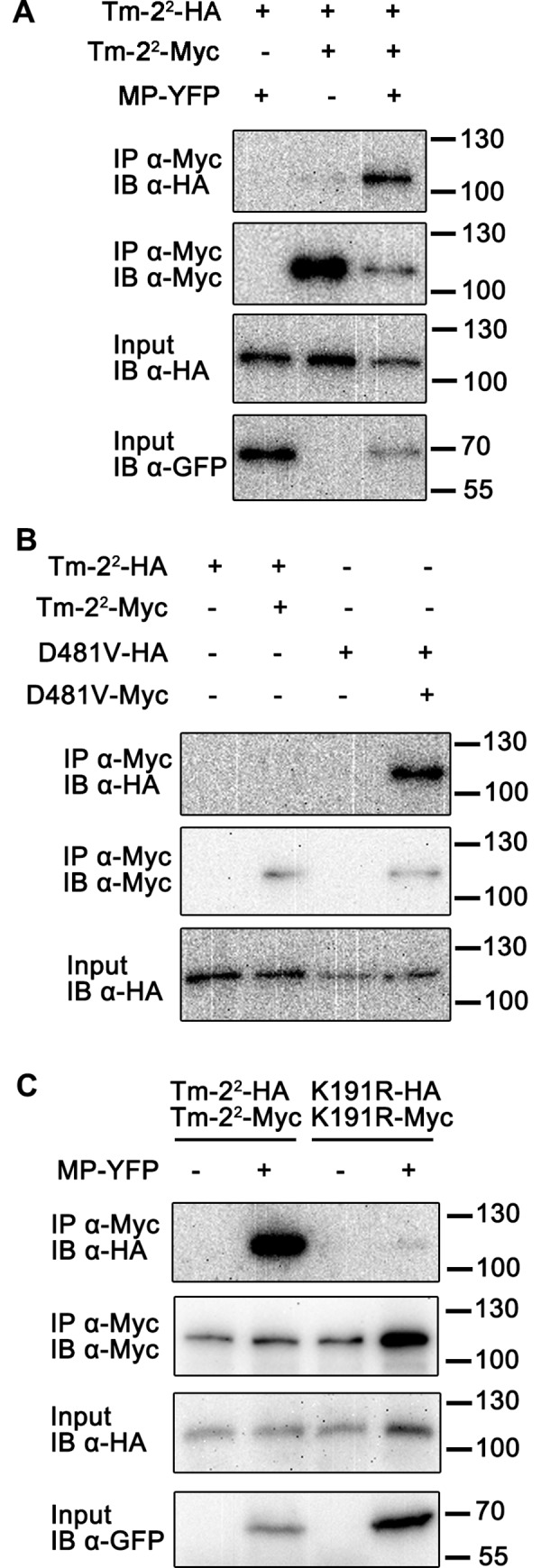
Tm-2^2^ self-associates upon activation. (A) Self-association of Tm-2^2^ was induced by TMV MP effector. (B) Tm-2^2^ (D481V) auto-active mutant self-associated in the absence of TMV MP. (C) Functional P-loop motif was required for TMV MP-induced Tm-2^2^ self-association. Indicated protein samples were subjected to anti-Myc immunoprecipitation (IP), followed by immunoblotting (IB) using the indicated antibodies. Protein molecular weight (kDa) marker is indicated on the right.

Many plant NLRs self-associate in the absence of effectors [19, 20, 33–36], but only a few exhibit effector-dependent oligomerization [14, 25, 32]. We examined whether Tm-2^2^ NLR exhibited effector-dependent self-association. For this, we transiently co-expressed Myc- and HA-tagged Tm-2^2^ in the absence or presence of YFP-tagged TMV MP in *Nicotiana benthamiana* and performed co-immunoprecipitation (co-IP) assays. Tm-2^2^-HA was co-expressed with MP-YFP as a negative control. The infiltrated leaves were collected when slight cell death began to occur at 23 hours post infiltration (hpi). In the absence of MP, Tm-2^2^-HA barely co-immunoprecipitated with Tm-2^2^-Myc, while in the presence of MP, Tm-2^2^-HA co-immunoprecipitated with Tm-2^2^-Myc (Fig 1A). These results indicate that viral MP promotes self-association of Tm-2^2^.

We have reported that a Tm-2^2^ auto-active mutant (D481V) contains a mutation in the MHD motif and triggers cell death in the absence of viral MP perhaps due to the conformational switch to an active status [48]. To test whether auto-active Tm-2^2^ can self-associate, we co-expressed the Tm-2^2^ (D481V) mutant with Myc and HA tags in *N*. *benthamiana* and performed co-IP assays. Samples were collected when slight cell death became visible. Indeed, we detected the self-association of the Tm-2^2^ auto-active mutant (Fig 1B), suggesting that the self-association of Tm-2^2^ is correlated with its activation.

In contrast to the MHD motif mutant, a mutation (K191R) in the conserved P-loop motif inhibits the function of Tm-2^2^ [48]. NLR P-loop mutants cannot bind nucleotide and fail to induce cell death [52, 53]. We next tested whether an intact P-loop motif is required for Tm-2^2^ self-association. In the presence of MP, HA-tagged Tm-2^2^ (K191R) failed to co-immunoprecipitate with Myc-tagged Tm-2^2^ (K191R), whereas the effector induced self-association of wild-type Tm-2^2^ (Fig 1C). Similarly, mutation of the conserved R291 residue in the RNBS-B motif of NB-ARC to alanine (R291A) inhibited MP-dependent Tm-2^2^-mediated self-association and cell death (S1 Fig). These results indicate that nucleotide binding is also important for Tm-2^2^ function and self-association.

### Domain interactions in Tm-2^2^

**Fig 2:**
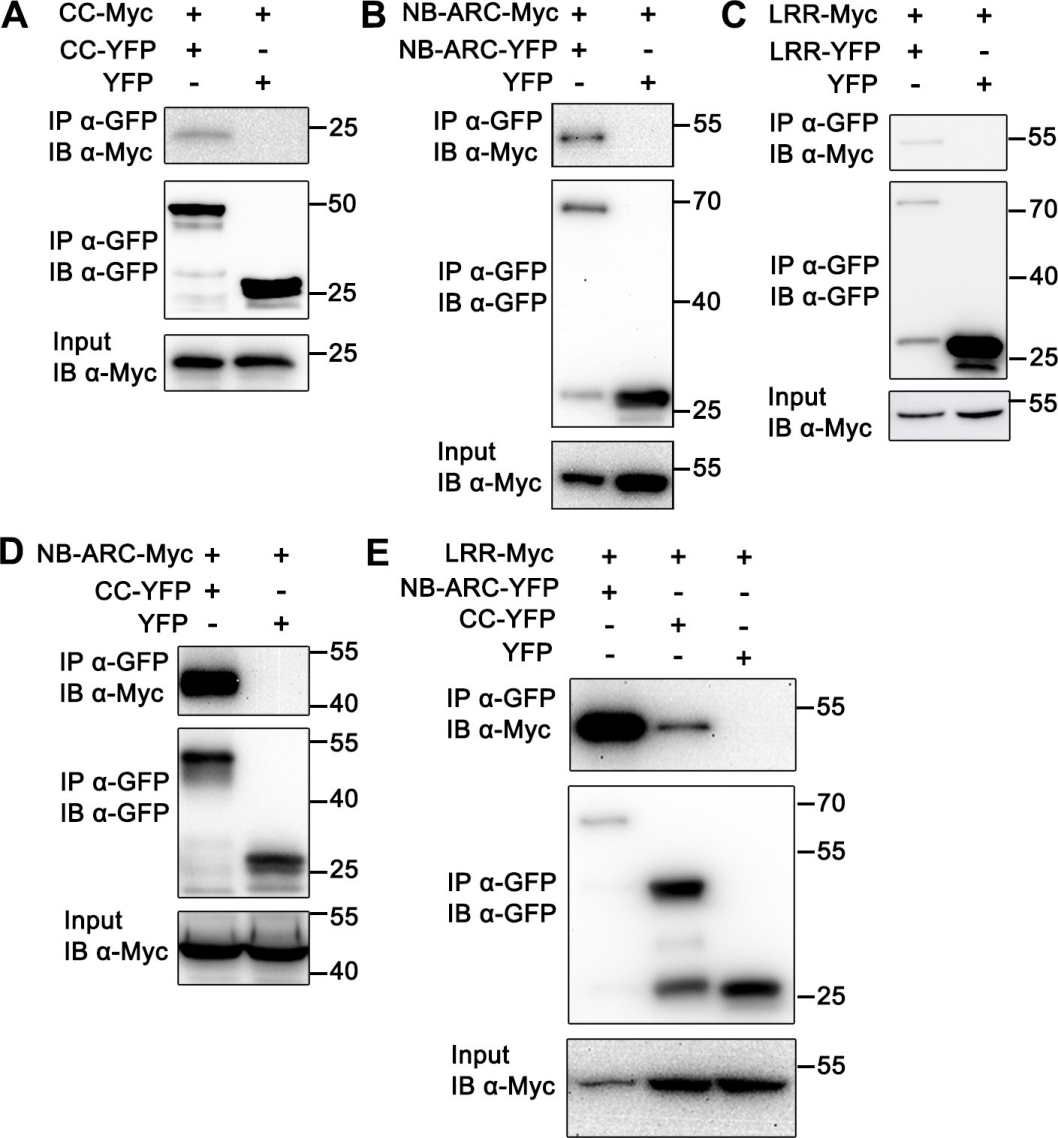
Domain interactions of Tm-2^2^. Co-IP experiments were performed to detect the self-association of CC (A), NB-ARC (B), or LRR domain (C). CC interacted with NB-ARC (D), LRR interacted with CC and NB-ARC (E). All the constructs were transiently expressed in *N*. *benthamiana* for 48 hours, and leaves were collected for co-IP by using anti-GFP beads. Co-IP samples were detected by IB with anti-Myc or anti-GFP antibody.

To further investigate Tm-2^2^ self-association, we tested whether the isolated domains of Tm-2^2^ can self-associate. For this, we expressed the Tm-2^2^ CC (aa 1–141), NB-ARC (aa 141–492) and LRR (aa 493–861) domains, fused to C-terminal YFP, Myc or HA tags in *N*. *benthamiana* for subsequent co-IP assays. The CC domain of several CC-NLR proteins has been shown to self-associate, and self-association is essential for their function [20, 34, 35]. Surprisingly, Tm-2^2^-CC-HA did not associate with Tm-2^2^-CC-Myc, although we could detect self-association of the RPM1 CC domain as described previously [34] (S2A Fig). However, we did detect weak Tm-2^2^ CC self-association when CC-YFP was used in co-IP assays with CC-Myc (Fig 2A), suggesting that Tm-2^2^ CC may have a weak or transient self-association ability. The NB-ARC domain self-associated in co-IP assays (Fig 2B and S2B Fig). For the LRR domain, LRR-Myc associated with LRR-YFP and LRR-HA (Fig 2C and S2C Fig). These findings indicate that more than one domain may mediate self-association of Tm-2^2^.

We also examined potential interactions among three Tm-2^2^ domains by co-IP assays. The data showed that CC-YFP co-immunoprecipitated with NB-ARC-Myc and LRR-Myc, and NB-ARC-YFP interacted with LRR-Myc (Fig 2D and 2E). These results suggest that multiple domain interactions regulate Tm-2^2^ function, and keep Tm-2^2^ in an inactive state before activation, similar to the inactive structure of ZAR1 maintained by contacts between multiple domains [15].

### Tm-2^2^ CC domain induces cell death when it is tethered to the PM

The CC domain of some cytosolic CC-NLRs has been shown to function as the signaling domain [19, 21, 35]. However, it is not clear which domain is the signaling domain for plasma membrane-localized NLRs. Overexpression of individual domains of Tm-2^2^ failed to induce cell death [48]. Considering that Tm-2^2^ functions at the PM, and that individual domains cannot efficiently be localized to the PM as is full-length Tm-2^2^ [48], we hypothesized that PM localization is indispensable for Tm-2^2^-mediated cell death.

**Fig 3:**
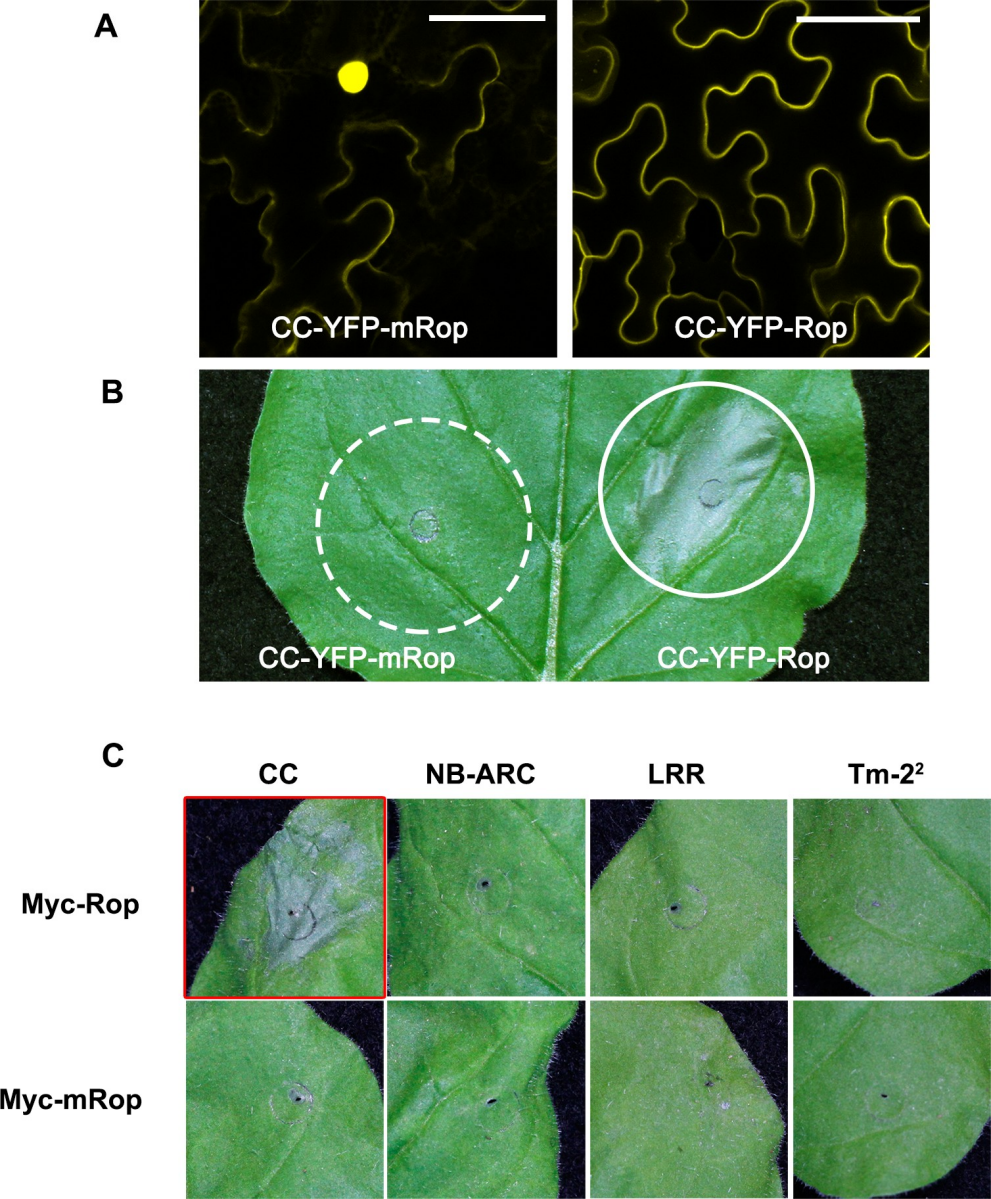
Tm-2^2^ CC domain triggers cell death upon PM localization. (A) The confocal images of CC-YFP-mRop and CC-YFP-Rop. Bar = 50 μm. (B) Only CC-YFP-Rop induced cell death in *N*. *benthamiana* leaves. Solid line circle indicates cell death; dashed line circle indicates no cell death at the infiltrated region. (C) Cell death phenotype of Tm-2^2^ or its domains fused to Myc-Rop or Myc-mRop. Only CC domain fusion with Myc-Rop induced cell death. The red box indicates cell death triggered by the fusion protein. The cell death phenotypes were photographed at 3 days post infiltration (dpi).

To identify the signaling domain of Tm-2^2^, individual domains were expressed fused C-terminally to the Myc epitope tag and the S-acylation PM association motif from *Arabidopsis* AtRop10 (named Rop-tag) that we have used previously to target proteins to the PM [48, 54]. We transiently expressed these proteins in *N*. *benthamiana* and examined their subcellular localization and their ability to induce cell death. Cellular fractionation assays showed that Rop-tag worked efficiently to tether all fusion proteins to the membrane fraction (S3 Fig). In addition, confocal microscopy observations showed that CC-YFP-Rop localized to PM, while CC with a mutated Rop-tag (CC-YFP-mRop) localized in the cytosol and nucleus (Fig 3A). Interestingly, while Tm-2^2^ CC-YFP-Rop and CC-Myc-Rop induced weak cell death in *N*. *benthamiana* leaves, CC-Myc-mRop and CC-YFP-mRop that failed to localize to the PM did not trigger cell death (Fig 3B and 3C). Tm-2^2^ NB-ARC or LRR domains failed to induce cell death, with either the Rop or mRop motifs (Fig 3C). Immunoblotting confirmed expression of all fusion proteins (S4 Fig). Cell death induced by CC-Myc-Rop was visible earliest at 34 hpi (hours post infiltration) compared to co-expression of full-length Tm-2^2^ with TMV MP that was visible at approximately 23 hpi. These findings demonstrate that the CC domain functions as a signaling domain for Tm-2^2^, and that PM localization is essential for its function.

### Self-association of Tm-2^2^ CC domain is important for inducing cell death

**Fig 4:**
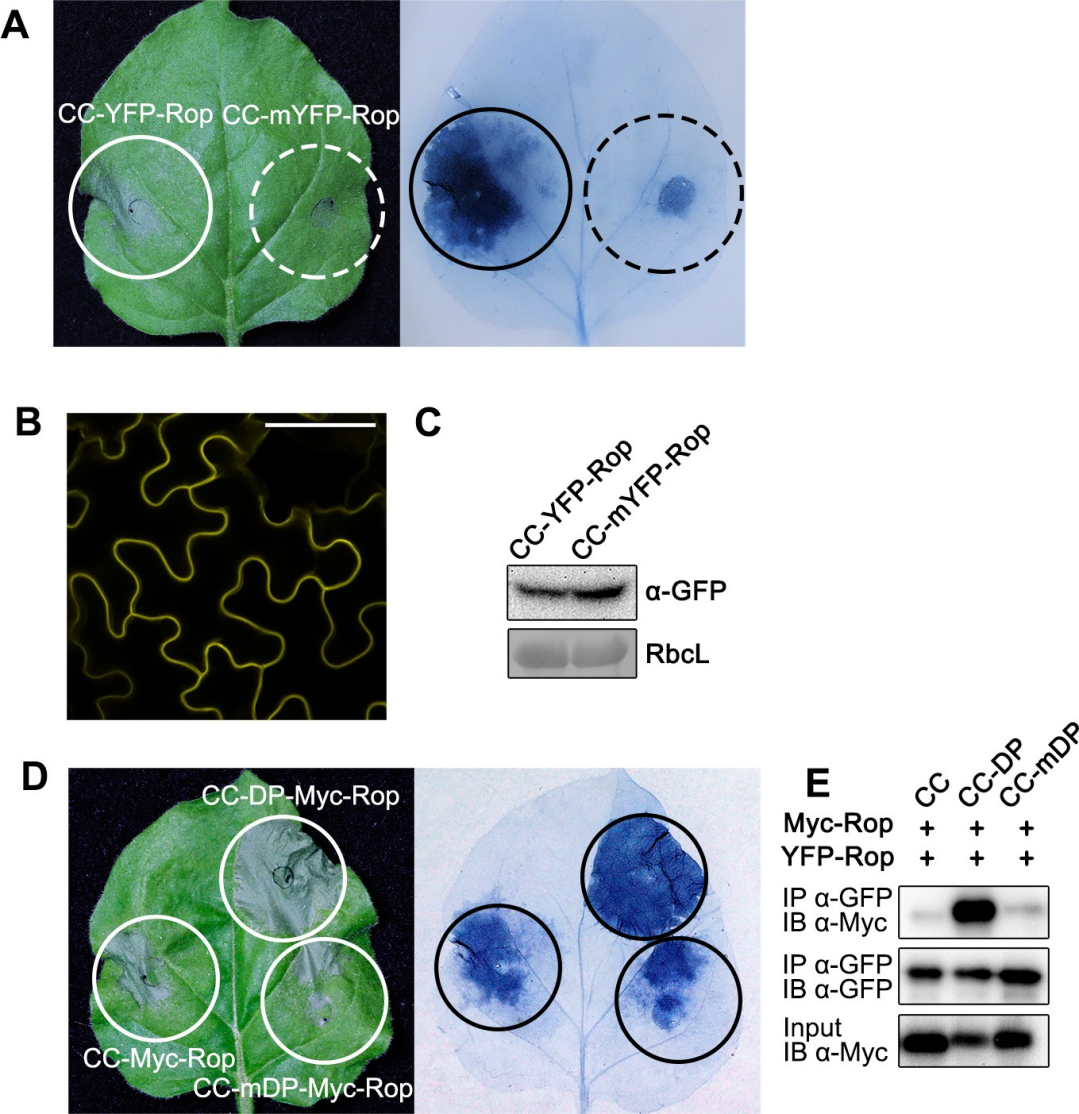
Enhanced self-association promotes Tm-2^2^ CC-mediated cell death. (A) Cell death mediated by CC fused with YFP-Rop or monomeric YFP-Rop (mYFP-Rop). The leaves were stained by trypan blue to facilitate observation of cell death. (B) The confocal images of CC-mYFP-Rop. Bar = 50 μm. (C) The protein levels of different fusion proteins. Rubisco L (RbcL) was stained by Ponceau S as loading control. (D) Cell death mediated by CC fused with dimerization peptide (DP) or mutant DP. CC fused to DP exhibited stronger cell death than CC fused to mDP. (E) DP enhanced the self-association of CC fusion proteins. Myc-Rop or YFP-Rop tagged CC fusion proteins were transiently expressed in pair. Proteins extracted from leaves were subjected to IP with anti-GFP beads, followed by IB using anti-Myc or anti-GFP antibody.

We tested the correlation between Tm-2^2^ CC self-association and CC-mediated cell death. Since the YFP tag used in the CC-YFP-Rop fusion protein has the ability to form weak dimers, and the dimerization of YFP can be blocked by the A206K mutation [38, 55], we genetically fused CC to the YFP A206K mutant (mYFP). CC-mYFP-Rop localized at the PM but triggered weaker cell death than CC-YFP-Rop (Fig 4A and 4B), while their protein levels were similar (Fig 4C). These data suggest that dimerization promotes Tm-2^2^ CC-mediated cell death.

Since Tm-2^2^ CC self-associates weakly, we enhanced its self-interaction by fusing it to a strong self-association motif. A 7-aa peptide called dimerization peptide (DP, EFLIVKS) induces self-association and a mutant form (mDP, EFLKVKS) fails to induce self-association [56]. We generated and transiently expressed CC-DP-Myc/YFP-Rop and CC-mDP-Myc/YFP-Rop in *N*. *benthamiana* to test the effect of DP on Tm-2^2^ CC self-association and cell death. Interestingly, CC fused to DP, but not mDP, enhanced CC-mediated cell death (Fig 4D). Co-IP assays confirmed that the DP significantly enhanced the self-association of Tm-2^2^ CC, while the mDP did not improve self-association (Fig 4E). These results suggest that self-association ability of Tm-2^2^ CC is correlated with cell death level. Together, these results indicate that self-association is required for the function of CC domain, and stronger self-association results in enhanced cell death.

### The NB-ARC domain regulates the activation of the CC domain

**Fig 5:**
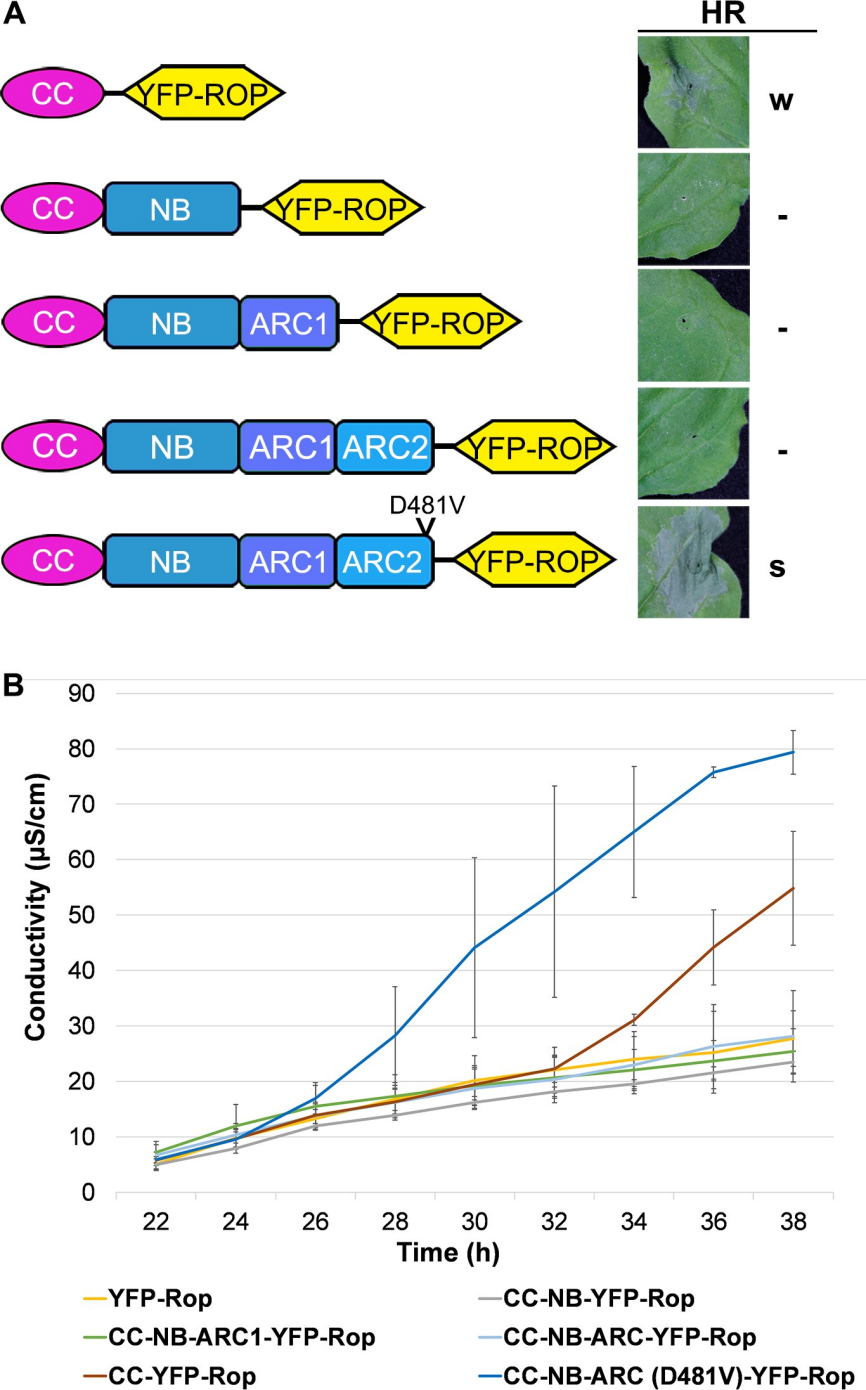
Tm-2^2^ NB-ARC domain regulates CC-mediated cell death. (A) Cell death mediated by CC with different NB-ARC extensions. Different YFP-Rop tagged CC extension constructs are shown schematically on the left side. All constructs were agro-inoculated into *N*. *benthamiana* leaves, and the cell death phenotypes were photographed at 3 dpi. “S” indicates strong cell death; “W” indicates weak partial cell death; “-” indicates no cell death observed. (B) Ion leakage caused by different constructs was measured at different time after agro-inoculation. The error bars indicate the standard deviation from 3 replicates. The experiment was performed at least three times with similar results.

Since full-length Tm-2^2^ is inactive before MP recognition, and the CC domain alone is sufficient to induce cell death, we hypothesized that other domains in Tm-2^2^ may regulate the CC domain during the activation of full-length Tm-2^2^. To investigate how the CC domain is regulated, we tested extended CC domain proteins containing additional NB-ARC motifs for the ability to induce cell death using an ion leakage assay. All of the YFP-Rop tagged fusions expressed in *N*. *benthamiana* localized at the PM (S5 Fig). The CC domain extended with the NB (aa 1–293), NB-ARC1 (aa 1–390), and NB-ARC1-ARC2 (NB-ARC) (aa 1–492) motifs failed to induce cell death (Fig 5 and S5 Fig), suggesting that NB-ARC domain inhibits the function of the CC domain.

Further, we generated a CC-NB-ARC molecule containing the auto-activating (D481V) mutation. Surprisingly, CC-NB-ARC (D481V)-YFP-Rop exhibited a stronger cell death than CC-YFP-Rop (Fig 5). Our findings indicate that the ADP-bound NB-ARC domain prevents CC from activation. Since the mutation in the MHD motif promotes preferential binding to ATP rather than ADP [7, 10], (d)ATP binding to NB-ARC domain releases the suppression of the CC domain and switches CC to the active state to enhance cell death.

### Self-association via the NB-ARC domain is necessary for CC-NB-ARC (D481V) and Tm-2^2^/MP mediated cell death

**Fig 6:**
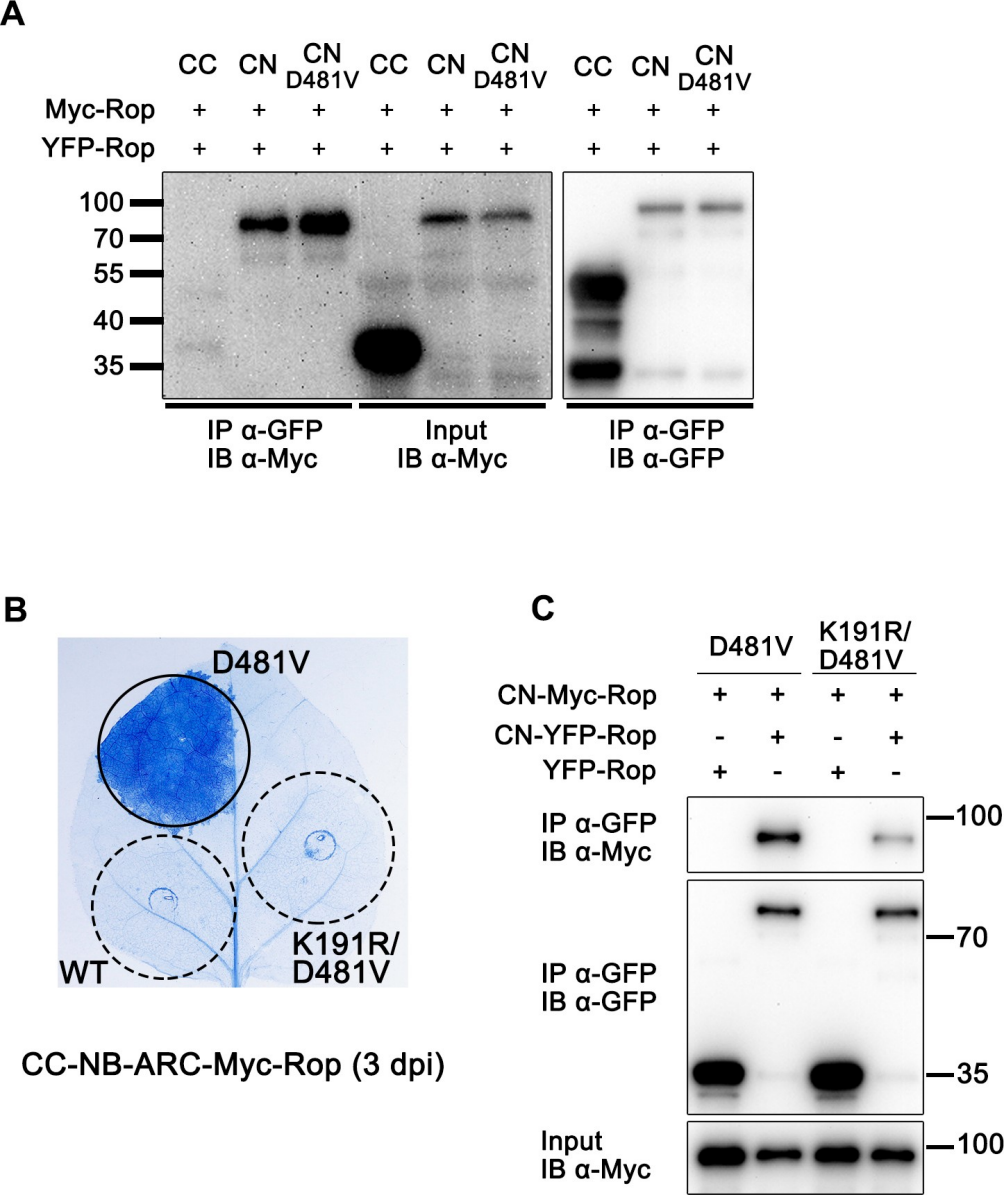
Nucleotide binding state switches CC-NB-ARC activation. (A) Self-association ability of CC, CC-NB-ARC or CC-NB-ARC (D481V). All domains were fused with Myc-Rop or YFP-Rop. CN stands for CC-NB-ARC. After co-expression, protein extracts were subjected to IP with anti-GFP beads, followed by IB with anti-GFP or anti-myc antibody. (B) and (C), K191R mutation repressed cell death signaling and impaired self-association of CC-NB-ARC (D481V)-Rop. Indicated protein samples were subjected to IP with anti-GFP beads, followed by IB with anti-Myc or anti-GFP antibody.

Since the MHD mutation D481V enabled CC-NB-ARC to induce cell death, we investigated how this mutation regulated CC domain function. Considering that stronger self-association helps CC function (Fig 4), we hypothesized that the D481V mutation may enhance the self-association between CC-NB-ARC proteins, thus leading to stronger CC self-association. Co-IP assays showed that self-association of wild-type CC-NB-ARC or CC-NB-ARC (D481V) was much stronger than that of the isolated CC domain (Fig 6A). However, CC-NB-ARC (D481V)-Rop only slightly enhanced self-association compared to wild-type CC-NB-ARC-Rop (Fig 6A). These results suggest that the stronger cell death induced by CC-NB-ARC (D481V)-Myc-Rop could correlate with its stronger self-association, but the self-association of CC-NB-ARC fusion proteins may not be the only factor that affects their function.

Based on previous studies [9–12, 14, 52, 53], the D481V mutation facilitates (d)ATP binding, while the loss-of-function P-loop mutation K191R and RNBS-B mutation R291A inhibit (d)ATP binding. Introduction of the K191R mutation into CC-NB-ARC (D481V)-Myc-Rop abolished cell death (Fig 6B). Further, the co-IP assay showed that K191R not only blocked cell death, but also impaired the self-interaction of CC-NB-ARC (D481V)-Rop (Fig 6C). Similarly, the R291A mutation also dampened cell death and self-association of CC-NB-ARC (D481V)-Rop (S6 Fig). Moreover, co-IP assays showed that both the K191R and R291A mutations impaired the self-association ability of NB-ARC (D481V) (S7 Fig), consistent with their effects on the self-association of CC-NB-ARC (D481V) (Fig 6C and S6B Fig) and full-length Tm-2^2^ (Fig 1C and S1B Fig). These results indicate that the nucleotide binding state of NB-ARC is crucial for the activation and self-association of both the Tm-2^2^ CC-NB-ARC and full-length proteins.

**Fig 7:**
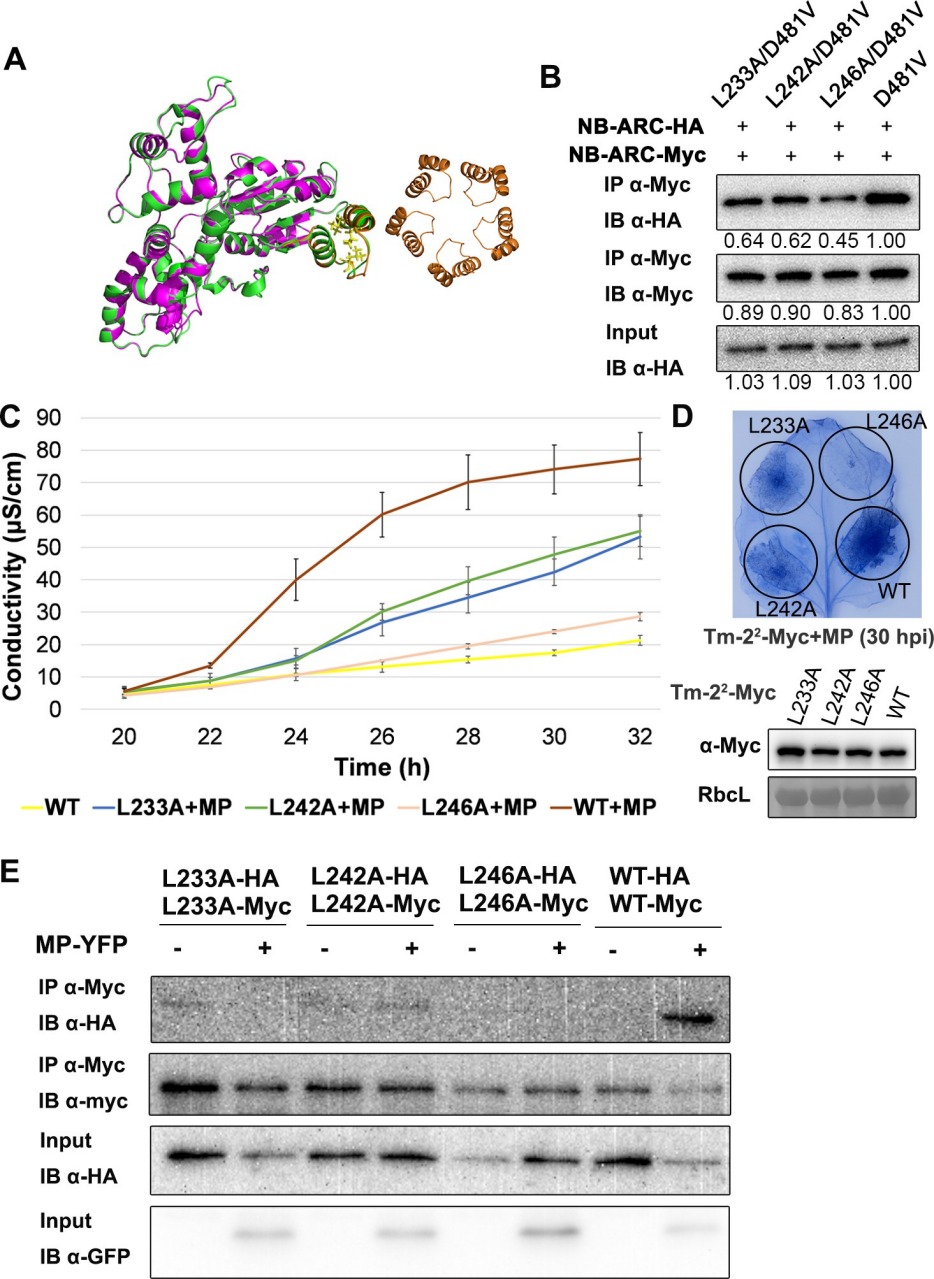
Tm-2^2^ requires NB-ARC self-association for its function. (A) Structure modeling of Tm-2^2^ NB-ARC domain. Green, the predicted structure of Tm-2^2^ NB-ARC domain; purple, the structure of ZAR1 (PDB ID: 6J5T); Orange, helix-loop-helix located at the center of ZAR1 inter-subunit-interface. Right orange ring shows the indicated helix-loop-helix motifs among the ZAR1 pentamer center [14]. (B) Potential oligomer surface mutations L233A, L242A and L246A weakened the self-association of NB-ARC (D481V). Numbers below the panel indicate the relative intensity analyzed by ImageJ. Indicated protein samples were subjected to IP with anti-Myc beads, followed by IB with anti-Myc or anti-HA antibody. (C) Ion leakage measurement showed L233A, L242A and L246A weakend full-length Tm-2^2^ mediated cell death in the presence of TMV MP. The error bar indicates the standard deviation from 3 technical repetitions. The experiment was performed at least three times with similar results. (D) Trypan blue staining of infiltrated leaves collected at 30 hpi showed that for full-length proteins, L233A, L242A and L246A mutations weakened the cell death triggered by Tm-2^2^/MP. The expression of Tm-2^2^ and its mutants were detected by anti-Myc antibody. RbcL was stained by Ponceau S as loading control. (E) L233A, L242A and L246A mutations weakened TMV MP induced full-length Tm-2^2^ self-association. Indicated protein samples were subjected to IP with anti-Myc beads, followed by IB with anti-Myc, anti-HA or anti-GFP antibody.

The NB-ARC domains of both CED-4 and Apaf-1 induce homo-oligomerization that is critical for their functions, and the first structure of a plant NLR, ZAR1, also indicates an essential role for the NB-ARC in resistosome oligomerization. Since the Tm-2^2^ NB-ARC domain self-associates (Fig 2B and S2B Fig), we tested whether the self-association of Tm-2^2^ NB-ARC domain was required for cell death mediated by CC-NB-ARC (D481V)-Myc-Rop and Tm-2^2^. The structure of Tm-2^2^ NB-ARC domain was predicted using I-TASSER (https://zhanglab.ccmb.med.umich.edu/I-TASSER/) based on the structure of ZAR1. The helix-loop-helix located at the center of the inter-subunit-interface is required for NB-ARC oligomerization in ZAR1 and CED-4 complexes [14, 57], and a similar structure was identified in Tm-2^2^ NB-ARC through structural modeling (Fig 7A). Three exposed residues (L233, L242 and L246) in the helix-loop-helix were identified in the Tm-2^2^ NB-ARC domain. We generated alanine (A) substitutions at residues L233, L242, and L246 in NB-ARC (D481V), and found that these three mutations weakened the self-association of NB-ARC (D481V) (Fig 7B). We also introduced these three mutations into CC-NB-ARC (D481V)-Myc-Rop, and found that these three mutants caused less cell death than CC-NB-ARC (D481V)-Myc-Rop (S8A Fig) after expression in *N*. *benthamiana* leaves. The L233A and L242A mutations delayed cell death mediated by CC-NB-ARC (D481V)-Myc-Rop by about 10 h, although these two mutants did eventually induce macroscopic HR, and the L246A mutation strongly blocked cell death. Co-IP assays demonstrated that the three mutations indeed disturbed the self-association of CC-NB-ARC (D481V)-Rop (S8C Fig). We further introduced the three mutations into full length Tm-2^2^. Ion leakage measurement and trypan blue staining showed that all three mutants delayed cell death induction in the presence of TMV MP (Fig 7C and 7D), although they sometimes achieved similar cell death intensity as Tm-2^2^ WT at 2 dpi. Further, these three mutations also weakened the MP-triggered self-association of Tm-2^2^ (Fig 7E). These results suggest that self-association of NB-ARC domain is important for cell death induction by Tm-2^2^.

## Discussion

In this study, we found that Tm-2^2^ exhibits effector-dependent self-association and that the CC domain is the signaling domain. Further, the Tm-2^2^ CC domain requires PM localization and self-association mediated by the NB-ARC domain for its function. To our knowledge, our findings described here constitute the first report on the signaling domain of a PM-localized plant NLR immune receptor and its mechanism of action.

### Tm-2^2^ requires effector-dependent self-association for its function

Animal NLR proteins usually exhibit elicitor-induced oligomerization [2]. Based on structural studies, mammalian NLRC4 is in the monomeric and auto-inhibited state, and binds ADP in the absence of pathogen [58]. NLR protein NAIP act as sensors for bacterial protein ligands. Upon pathogen recognition, activated NAIP binds to NLRC4 to create an interaction surface for NLRC4 oligomerization leading to assembly of a wheel-shaped oligomer [31]. There is currently no unifying model in plants to illustrate the relationship between oligomerization and NLR function. Some plant NLR domains or full-length proteins have been shown to self-associate and some self-associate independent of activation [34]. Pre-oligomeric NLRs may function as a trap to detect pathogen effectors, such as Prf and its accessory protein Pto [36, 59]. Among those plant NLRs studied, only a few have been reported to form effector-induced oligomers [14, 25, 32]. However, the roles and mechanisms of plant NLR oligomerization remain largely unknown.

In this study, we found that Tm-2^2^ can self-associate after activation. Tm-2^2^ self-association is triggered either by effector recognition or incorporation of an auto-activating mutation into the conserved MHD motif (Fig 1A and 1B). Further, the P-loop and RNBS-B motifs are essential for Tm-2^2^ self-association (Fig 1C and S1B Fig), which is consistent with previous studies on N, RPP1 and ZAR1 [14, 25, 32]. Our results suggest that Tm-2^2^ self-association requires its binding to (d)ATP, and is necessary for triggering the downstream signaling. Considering the pentamer structure of ZAR1 and the induced self-association model, Tm-2^2^ may also form oligomers upon activation.

Previously reported plant NLR oligomerization events operate via different domains: N depends on its TIR domain, RPP1 needs multiple interaction interfaces, and ZAR1 relies on its CC and NB-ARC domains [14, 25, 32]. In this study, our results show that all three domains of Tm-2^2^ (CC, NB-ARC and LRR) have the ability to self-associate, suggesting that the induced oligomerization of Tm-2^2^ may involve multiple self-association surfaces, similar to RPP1.

### Tm-2^2^ requires oligomerization of its CC domain to induce cell death at the PM

The CC domains of several CC-NLRs have been reported to induce cell death, but these CC domains localize to the nucleus or cytosol [12, 20]. Some CC-NLRs are reported to function at the PM, but their signaling domain for triggering auto-active cell death is unknown [17, 34, 49, 50]. In a recent study, we found that expression of the Tm-2^2^ CC domain alone fails to target the protein to the PM and does not induce cell death [48]. In this study, we show that Tm-2^2^ CC triggers cell death in *N*. *benthamiana* once it is targeted to the PM via a heterologous S-acylation motif. These findings are consistent with the PM localization of full-length Tm-2^2^ [48]. Different CC-NLRs in diverse subcellular compartments may recruit different signaling components to induce cell death. Our study demonstrates the first minimal signaling effector domain for a PM-localized CC-NLR.

Several TIR domains of TIR-NLRs and CC domains of CC-NLRs are able to induce cell death, and their self-association is required for cell death [20, 40]. In this study, we found that Tm-2^2^ CC exhibited very weak self-association, and induced only a weak cell death phenotype. The self-association ability was correlated with the strength of cell death. GFP/YFP has weak dimerization ability, and fusion with GFP/YFP can force the fusion protein into a dimer [38, 55]. The TIR domain of RPP1 fused to GFP, but not mutated monomeric GFP, can induce cell death [38]. In this study, we also found that YFP fusion enhanced Tm-2^2^ CC induced cell death compared to fusions with monomeric mYFP (Fig 4A–4C). In addition, fusions with an artificial dimerization peptide strengthened the interaction between Tm-2^2^ CC domains and enhanced cell death (Fig 4D and 4E). These findings strongly support that Tm-2^2^ CC domain self-association is required for its function. Interestingly, CC-Myc-Rop, like CC-YFP-Rop, was able to induce stronger HR than CC-mYFP-Rop. It is possible that the large mYFP-tag hinders dimerization of the CC domain, thus leading to weaker CC-mediated cell death.

Tm-2^2^ requires its CC self-association, PM localization (in this study) and N-terminus exposure [48] for function, which is reminiscent of the ZAR1 model [14]. Once activated, ZAR1 increases its association with the PM and forms a pentameric structure in which CC self-associates and its extreme N terminus region forms a funnel-shaped structure. The activated ZAR1 pentamers may perturb PM integrity and/or ion homeostasis, leading to cell death and rapid expression of defense genes [14]. Similarly, assembly of Tm-2^2^ CC oligomers at the PM might also perturb PM integrity and/or ion homeostasis, or interact with other PM-localized signaling components to trigger cell death and defense.

### Tm-2^2^ NB-ARC regulates CC-mediated cell death

The limited self-association ability of Tm-2^2^ CC and the necessity of self-association for its function suggest that other domains could contribute to CC oligomerization. Interestingly, NB-ARC inhibited CC-mediated cell death (Fig 5) while CC-NB-ARC fragment strongly self-associates (Fig 6A), which suggests strong self-association is not sufficient for cell death induction. Similar to our findings, maize Rp1 CC-mediated cell death is also inhibited by its NB-ARC domain [35].

Different from the wild-type NB-ARC domain, NB-ARC (D481V) enhanced the CC-mediated cell death, indicating NB-ARC domain can act as a switch to regulate CC domain-mediated cell death. It will be interesting to investigate how D481V mutation positively regulates the function of CC-NB-ARC. We hypothesize that the D481V mutation may trigger the conformational changes to release NB-ARC inhibition of CC domain and strengthen the self-association of CC-NB-ARC for more effective CC function. This is partially supported from our observation that the D481V mutation can strengthen the self-association and cell death induction of CC-NB-ARC and CC (Figs 5 and 6A). Since the D481V mutation induced cell death but only slightly increased the self-association ability of CC-NB-ARC compared to wild-type (Figs 5 and 6A), other factors must be involved in NB-ARC regulation on CC domain. The nucleotide status of NB-ARC proteins is essential for stabilizing the active conformations of these proteins, for example, (d)ATP binding in the Apaf-1 apoptosome, ZAR1 resistosome and CED-4 apoptosome [14, 57, 60]. During formation of the ZAR1 resistosome, both CC and NB-ARC domains undergo remarkable structural changes after (d)ATP binding [14]. It is likely that for Tm-2^2^, the D481V mutation mimics the (d)ATP-bound state, while K191R and R291A destroy nucleotide binding and compromise cell death induction. (d)ATP binding to NB-ARC may trigger conformational changes in both the CC and NB-ARC domains, releasing NB-ARC-mediated repression on CC. Once the removal of this repression, (d)ATP-bound NB-ARC domains self-associate, thus help CC self-associate for downstream signaling. Through analyses of the predicted structure by mutagenesis, we found that three site mutations (L233A, L242A and L246A) can affect the self-association of active NB-ARC. Furthermore, these mutations disturbed the self-association and cell death mediated by both the CC-NB-ARC (D481V) and full-length Tm-2^2^ proteins (S8 Fig and Fig 7). These mutagenesis experiments support the important role of NB-ARC self-association in Tm-2^2^ CC mediated cell death. Accompanying the structural shift caused by D481V mutation, the interaction surfaces on NB-ARC that drive oligomer formation are exposed. Lastly, the force driving CC-NB-ARC (D481V)-mediated cell death may come mainly from switching the protein into the (d)ATP-bound state, in which structural changes relieve the NB-ARC repression of CC, and drive stronger self-association to trigger cell death and defense.

### Molecular domain interaction of Tm-2^2^

In animal NLR models, LRR functions as a repressor to inhibit the NLR from activation, and data on some plant NLRs do support this model. For RPS5, a CC-NB-ARC fragment is auto-active, while the first 4 LRRs are sufficient to hold the truncated protein inactive [17]. For Rx, the inhibitory interaction between CC-NB-ARC and LRR is released during effector recognition [61]. For Tm-2^2^, the LRR is required for recognition of effectors and recognition specificity [62]. In this study, we also found that LRR can interact with CC and NB-ARC, suggesting that LRR may also function as a repressor. Although the truncated Tm-2^2^ CC-NB-ARC fragment strongly self-associates (Fig 6A), the self-association of full-length Tm-2^2^ protein is barely detectable before activation (Fig 1A). Our findings may suggest that the Tm-2^2^ oligomerization surfaces in the resting state are blocked by LRR. Considering that CC-NB-ARC-Rop fails to induce cell death, NB-ARC may suppress CC function in the absence of MP. Due to the different self-association abilities of CC-NB-ARC and Tm-2^2^ in the absence of MP, the LRR domain in the resting state could provide another mechanism to inhibit CC auto-activity by suppressing oligomerization. In addition, co-IP assays showed that LRR possessed self-interaction ability (Fig 2C and S2C Fig), suggesting that LRR may also help Tm-2^2^ to oligomerize during activation of the full-length Tm-2^2^ protein.

### A model for Tm-2^2^ activation

**Fig 8:**
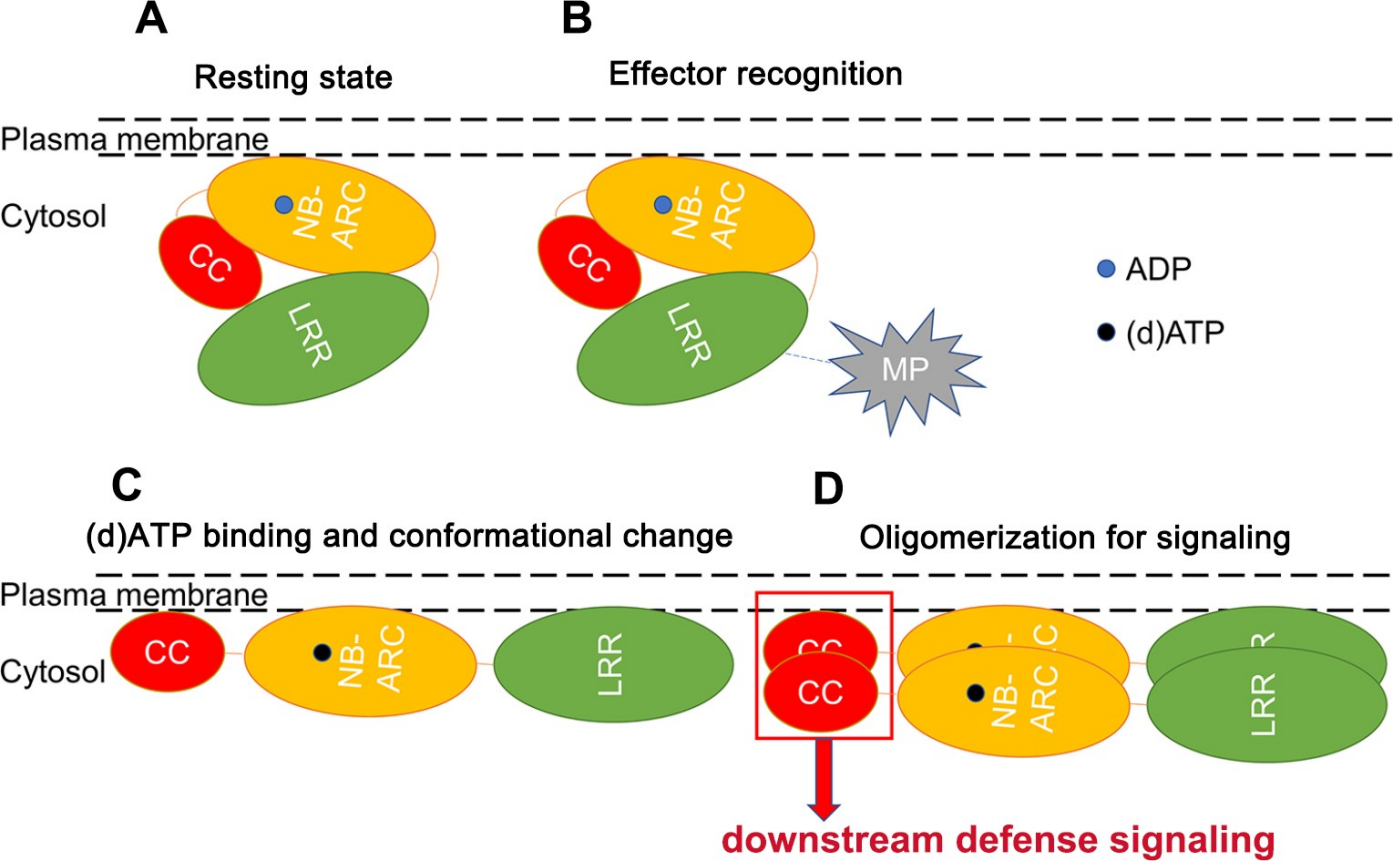
A model for Tm-2^2^ activation. (A) Tm-2^2^ localizes at the PM with multiple domain interactions. (B) LRR recognizes its effector MP through direct or indirect interaction. (C) Tm-2^2^ binds (d)ATP instead of ADP accompanied with conformational change. (D) At least two Tm-2^2^ molecules self-associate, thus leading to the self-association of CC domains for further defense signaling.

In the resting state, LRR interacts with CC and NB-ARC to inhibit Tm-2^2^ from auto-activation and self-association. ADP-bound NB-ARC interacts with the CC domain to repress auto-activation. During infection, LRR perceives viral MP, and de-represses the NB-ARC domain to switch ADP for (d)ATP, leading to important conformational changes. (d)ATP binding releases suppression of CC domain and promotes stronger self-association of the NB-ARC domain. Meanwhile, self-association of NB-ARC enhances the dimerization or oligomerization of CC domain. Homo-dimerized/oligomerized CC domain at the PM activates downstream defense signaling (Fig 8). It is worthy to mention that cell death and resistance are not entirely equivalent. This study deals exclusively with induction of cell death. It needs further investigation whether this model is applied to the induction of virus resistance.

## Materials and methods

### Plant material and plasmids

*N*. *benthamiana* plants were grown at 25°C under a 16-h-light/8-h-dark cycle. All T-DNA expression vectors were generated by ligation-independent cloning (LIC) method [48]. pLIC-Myc, pLIC-HA, pLIC-YFP vectors were described and pLIC-Myc-Rop, pLIC-Myc-mRop, pLIC-YFP-Rop and pLIC-YFP-mRop vectors were constructed in the same way as described [48]. In these constructs, gene expression was driven by CaMV 35S promoter. Before LIC cloning, LIC basic vectors were digested with *Apa*I and treated with T4 DNA polymerase in the presence of dTTP. *Tm-2*^*2*^ mutants were generated by overlapping PCR. All PCR products treated with T4 DNA polymerase in the presence of dATP were ligated into the treated LIC basic vectors as described [63]. Primers used for plasmid construction in this study are listed (S1 Table).

### Transient protein expression in *N*. *benthamiana*

For *Agrobacterium tumefaciens*-mediated transient protein expression in *N*. *benthamiana*, Agrobacterium GV3101 strains containing the relevant expression vectors were cultivated overnight, pelleted, resuspended in infiltration buffer (10 mM MES, 10 mM MgCl_2_, and 200 μM acetosyringone, pH 5.6), and kept at room temperature for 2–6 h before infiltration into plant leaves. Usually, an optical density of OD_600_ for inoculation is adjusted to 1 for each single strain, while for the GV3101 strain expressing MP-YFP, OD_600_ = 0.5.

### Protein analyses and Co-IP

For protein analysis, target proteins were expressed for 22–48 h in *N*. *benthamiana* leaves and then extracted with a ratio of 1 g leaf tissues:2 mL Laemmli buffer [64]. For co-expression of Tm-2^2^ and MP, samples were usually collected at 22–24 hpi. For expression of CC-NB-ARC (D481V)-Rop, samples were usually collected at 24–26 hpi.

Co-IP assays were performed as described previously [65] with following modifications. Briefly, total protein from 1–2 g leaf tissues were extracted using prechilled 2.5 mL IP buffer per gram (10% [v/v] glycerol, 25 mM Tris-HCl, pH 7.5, 1 mM EDTA, 150 mM NaCl, 10 mM DTT, protease inhibitor cocktail, and 0.2–0.5% [v/v] NP-40). Protein extracts were incubated with corresponding beads for 3 h at 4°C. The beads were washed 5 times with prechilled IP buffer at 4°C and then boiled in 50 μL 2× Laemmli buffer.

Anti-Myc beads (Abmart) and GFP-trap beads (ChromoTek) were used for co-IP assays. All protein samples were analyzed by SDS-PAGE, immunoblotted with anti-Myc (Abmart), anti-GFP (ChromoTek), and anti-HA (Cell Signaling Technology) antibodies. Protein levels were detected using a SuperSignal West Pico PLUS Chemiluminescent Substrate or SuperSignal West Femto Maximum Sensitivity Substrate (Thermo).

### Confocal microscopy

The leaves expressing related proteins were collected at 24–36 hpi. Confocal imaging with an inverted Zeiss LSM 710 laser scanning microscope (laser: 514 nm, filter: 519–620 nm) was used for observing target protein localization from the lower leaf surface.

### Membrane fractionation

Plasma membrane protein isolation kit for Plants (Invent, SM-005-P) was used for membrane fractionation. After homogenized by buffer A, plant tissues pass through a specialized filter cartridge with a zigzag path. After discard of the cartridge, the remaining homogenates were re-suspended, and then centrifuged at 700 X g for discarding the pellet containing nuclei, chloroplasts and larger debris. Total protein for immunoblotting was sampled from the supernatant. After new round of centrifuge at 16,000 X g, the original supernatant (total protein) can be divided into two parts: new supernatant (soluble fraction) and pellet (microsomal membrane fraction). Anti-H^+^-ATPase (Agrisera) was used for membrane protein indication.

### Trypan blue staining

*N*. *benthamiana* leaves were boiled for 5 min in a 2:1 mixture of ethanol and staining stock solution (mixture of 10 mL glycerol, 10 mL lactic acid, 10 mL phenol, 10 mL water, and 20 mg trypan blue). The leaves were then faded with 2.5 g/mL chloral hydrate solution.

### Electrolyte leakage measurement

Electrolyte leakage assays were performed as described previously [12] with following modifications. Agrobacterium suspensions were prepared as described above and infiltrated into *N*. *benthamiana* leaves. For every technical replicate, 6 leaf discs were collected using a puncher (disc area = 6 mm*6 mm) and floated on 4 mL of distilled water for 30 min. The distilled water was replaced by 4 mL fresh distilled water containing 0.001% Tween-20. The mixture of leaf disks and water was gently blended before every measurement. The electrolyte leakage was measured at room temperature for every 2 hours with FiveEasy Plus^TM^ FE38-Standard conductivity meter (METTLER TOLEDO). Means and error bars were calculated from three replicates per time point and construct.

## Supporting information

S1 Table Primers used in this study. (PDF)

S1 Fig R291 in Tm-2^2^ is crucial for MP-induced cell death and self-association of Tm-2^2^. (A) Tm-2^2^ (R291A) failed to induce cell death compared to WT in the presence of TMV MP. Myc-tagged Tm-2^2^ and Tm-2^2^ (R291A) were expressed with MP-YFP for cell death assays. The left picture was taken 2 dpi, and the right one represents the trypan blue staining of the same leaf. (B) Tm-2^2^ (R291A) disrupted the self-association of activated Tm-2^2^. Indicated protein samples were subjected to IP with anti-Myc bead. The markers of protein molecular weight (kDa) are indicated on the right. (C) Ion leakage caused by different Tm-2^2^ mutants was measured over time after infiltration. The error bar indicates the standard deviation from 3 technical repetitions. The experiment was performed at least three times with similar results.
(TIF)

S2 Fig Self-association of Tm-2^2^ domains. Fusion proteins with C-terminally HA or Myc tag were used to perform co-IP assays. All samples were subjected to IP with anti-Myc beads. (A) Tm-2^2^ CC-HA was not immunoprecipitated by Tm-2^2^ CC-Myc, but RPM1 CC-HA interacted with RPM1 CC-Myc in the same condition. (B) Tm-2^2^ NB-ARC-HA was immunoprecipitated by Tm-2^2^ NB-ARC-Myc. (C) Tm-2^2^ LRR-HA was immunoprecipitated by Tm-2^2^ LRR-Myc relative to empty control.
(TIF)

S3 Fig Fractionation of CC, NB-ARC and LRR fused to Rop or mRop motif. Cell lysates were separated to the soluble and microsomal membrane fractionations. T, total protein; S, soluble fraction; M, microsomal membrane fraction. H^+^-ATPase is the PM marker detected by IB, and RbcL is the soluble protein marker detected by Ponceau S.
(TIF)

S4 Fig Western blot of Rop or mRop fusion proteins. (A) Fusion proteins were probed with anti-Myc antibody. (B) Fusion proteins were probed with anti-GFP antibody.
(TIF)

S5 Fig Subcellular localization and expression level of YFP-Rop fusion proteins. (A) Confocal images showed YFP-Rop successfully tethered fusion proteins to the PM. (B) Protein levels were detected by anti-GFP antibody.
(TIF)

S6 Fig Importance of R291 in CC-NB-ARC (D481V)-Rop. (A) R291A mutation inhibited cell death mediated by CC-NB-ARC (D481V)-Myc-Rop. Pictures were taken at 3 dpi. (B) R291A disrupted the self-association of CC-NB-ARC (D481V)-Rop. All samples were subjected to IP with anti-GFP beads.
(TIF)

S7 Fig K191R and R291A disrupted the self-association of NB-ARC (D481V). Co-IP assay showed that both K191R and R291A disrupted the self-association of NB-ARC (D481V). All samples were subjected to IP with anti-Myc beads.
(TIF)

S8 Fig L233A, L242A and L246A disturbed the cell death phenotype and self-association of CC-NB-ARC (D481V)-Rop. (A) Trypan blue staining showed that L233A, L242A and L246A disturbed the cell death phenotype induced by CC-NB-ARC (D481V)-Myc-Rop. (B) The expression of CC-NB-ARC-Myc-Rop and its mutants were detected by anti-Myc antibody. RbcL was stained by Ponceau S as loading control. (C) Co-IP assay showed that L233A, L242A and L246A disturbed the self-association of CC-NB-ARC (D481V)-Rop. All samples were subjected to IP with anti-GFP beads.
(TIF)
