# Supplementary material for: Plant NLR immune receptor Tm-22 activation requires NB-ARC domain-mediated self-association of CC domain
Source: PLoS Pathog. 2020 Apr 27;16(4):e1008475. doi: 10.1371/journal.ppat.1008475 (PMC7205312; doi:10.1371/journal.ppat.1008475)
Supplement: S1 Table — (PDF) [file ppat.1008475.s001.pdf]

S1 Table. Primers used in this study.

For generating CC-LIC vector	Tm-2^2-LIC1-F	**CGACGACAAGACCGTGACC**ATGGCTGAAATTCTTCTTACATCAG
	CC-LIC2-R	**GAGGAGAAGAGCCGTCG**AACACAATCATCATTATTGTTAT
For mYFP (A206K)	mYFP-F	GCTACCAGTCCAAGCTGAGCAAAGAC
	mYFP-R	GTCTTTGCTCAGCTTGGACTGGTAGC
For CC-NB	NB-LIC2-R	**GAGGAGAAGAGCCGTCG**ACTATTTCGAGACGTGATTATCATTCTAC
For-CC-NB-ARC1	ARC1-LIC2-R	**GAGGAGAAGAGCCGTCG**ACATCCATCTTGAACTTTATGGCCC
For CC-DP	DP-LIC2-R	**GAGGAGAAGAGCCGTCG**AGACTTAACAATCAAAAATTC AACACAATCATCATTATTGTTAT
For CC-mDP	mDP-LIC2-R	**GAGGAGAAGAGCCGTCG**AGACTTAACCTTCAAAAATTC AACACAATCATCATTATTGTTAT
For R291A	R291A-F	GATAATCACGTCTGCAAATAGTAATGTAG
	R291A-R	CTACATTACTATTTGCAGACGTGATTATC
For L233A	L233A-F	CCAAACAAATTGGAGCTACGGAACAGAAAATG
	L233A-R	CATTTTCTGTTCCGTAGCTCCAATTTGTTTGG
For L242A	L242A-F	GAAAATGAAGGAAAATGCTGAGGACAACCTGCGATC
	L242A-R	GATCGCAGGTTGTCCTCAGCATTTTCCTTCATTTTC
For L246A	L246A-F	TGGAGGACAACGCACGATCACTCTT
	L246A-R	AAGAGTGATCGTGCGTTGTCCTCCA

Letters in bold indicate LIC1 or LIC2 adaptor sequences.
