# Supplementary figures and images for: Plant NLR immune receptor Tm-22 activation requires NB-ARC domain-mediated self-association of CC domain
Source: PLoS Pathog. 2020 Apr 27;16(4):e1008475. doi: 10.1371/journal.ppat.1008475 (PMC7205312; doi:10.1371/journal.ppat.1008475)

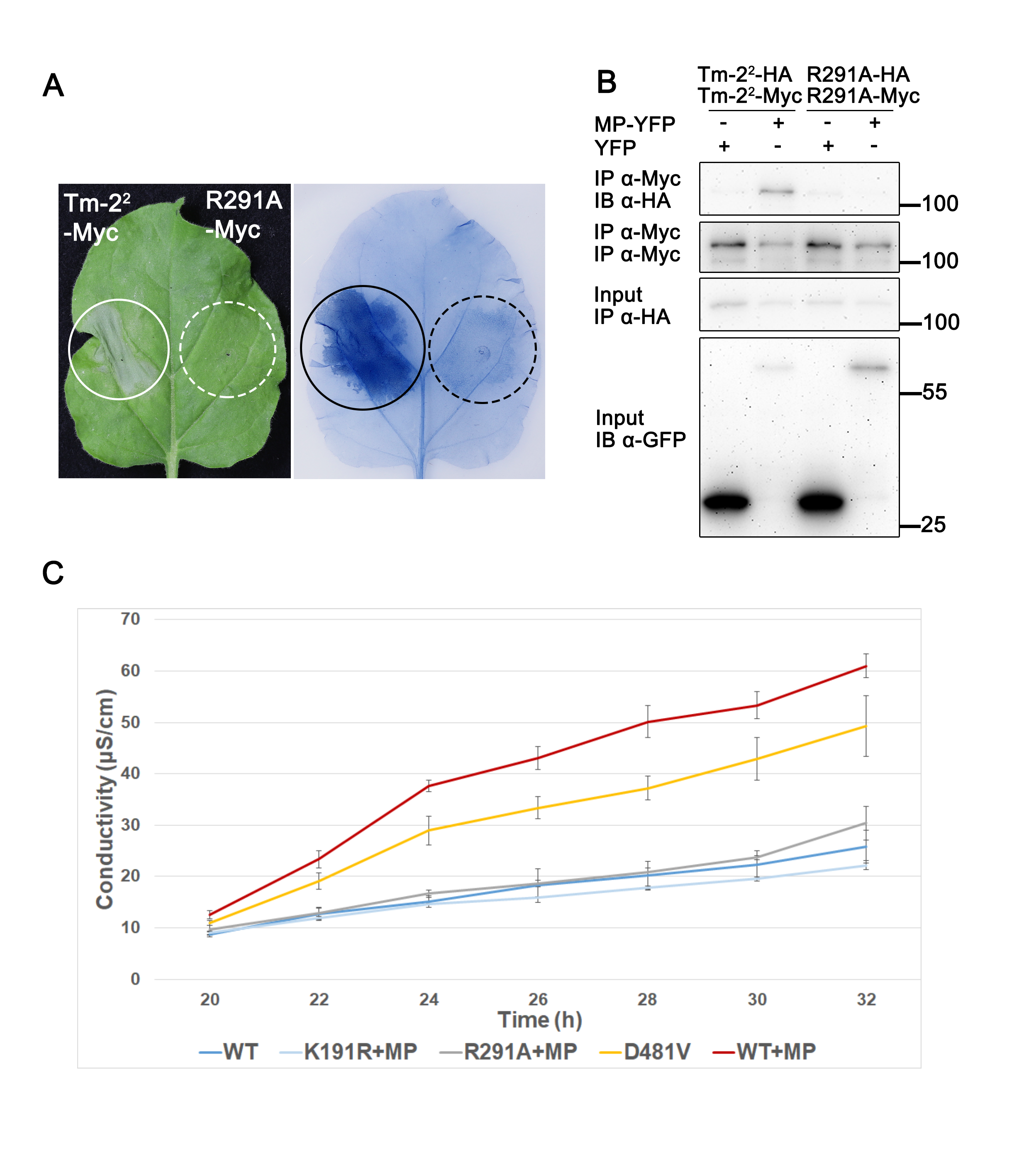

Supplement: S1 Fig — (A) Tm-22 (R291A) failed to induce cell death compared to WT in the presence of TMV MP. Myc-tagged Tm-22 and Tm-22 (R291A) were expressed with MP-YFP for cell death assays. The left picture was taken 2 dpi, and the right one represents the trypan blue staining of the same leaf. (B) Tm-22 (R291A) disrupted the self-association of activated Tm-22. Indicated protein samples were subjected to IP with anti-Myc bead. The markers of protein molecular weight (kDa) are indicated on the right. (C) Ion leakage caused by different Tm-22 mutants was measured over time after infiltration. The error bar indicates the standard deviation from 3 technical repetitions. The experiment was performed at least three times with similar results. (TIF) [file ppat.1008475.s002.tif]

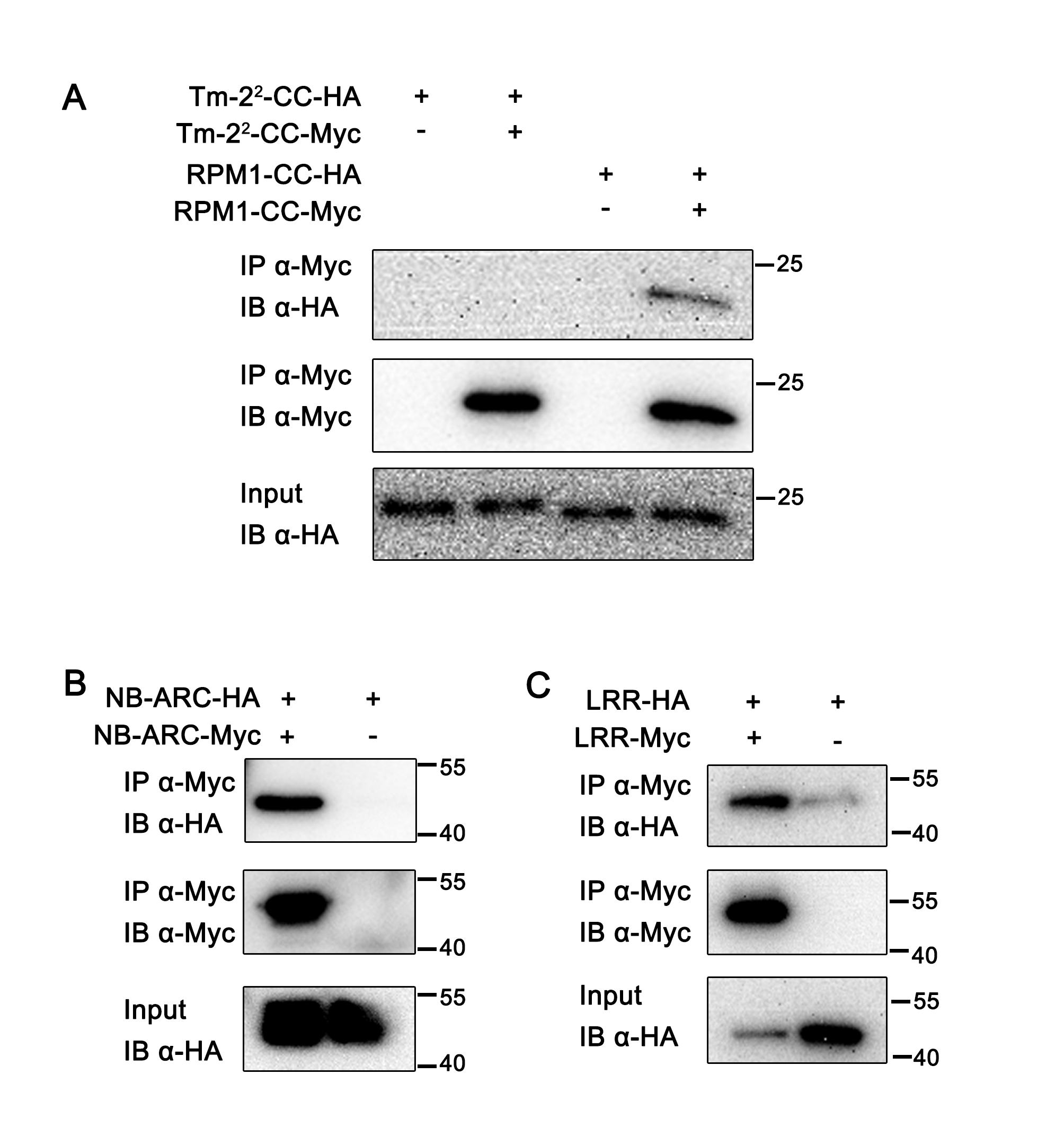

Supplement: S2 Fig — Fusion proteins with C-terminally HA or Myc tag were used to perform co-IP assays. All samples were subjected to IP with anti-Myc beads. (A) Tm-22 CC-HA was not immunoprecipitated by Tm-22 CC-Myc, but RPM1 CC-HA interacted with RPM1 CC-Myc in the same condition. (B) Tm-22 NB-ARC-HA was immunoprecipitated by Tm-22 NB-ARC-Myc. (C) Tm-22 LRR-HA was immunoprecipitated by Tm-22 LRR-Myc relative to empty control. (TIF) [file ppat.1008475.s003.tif]

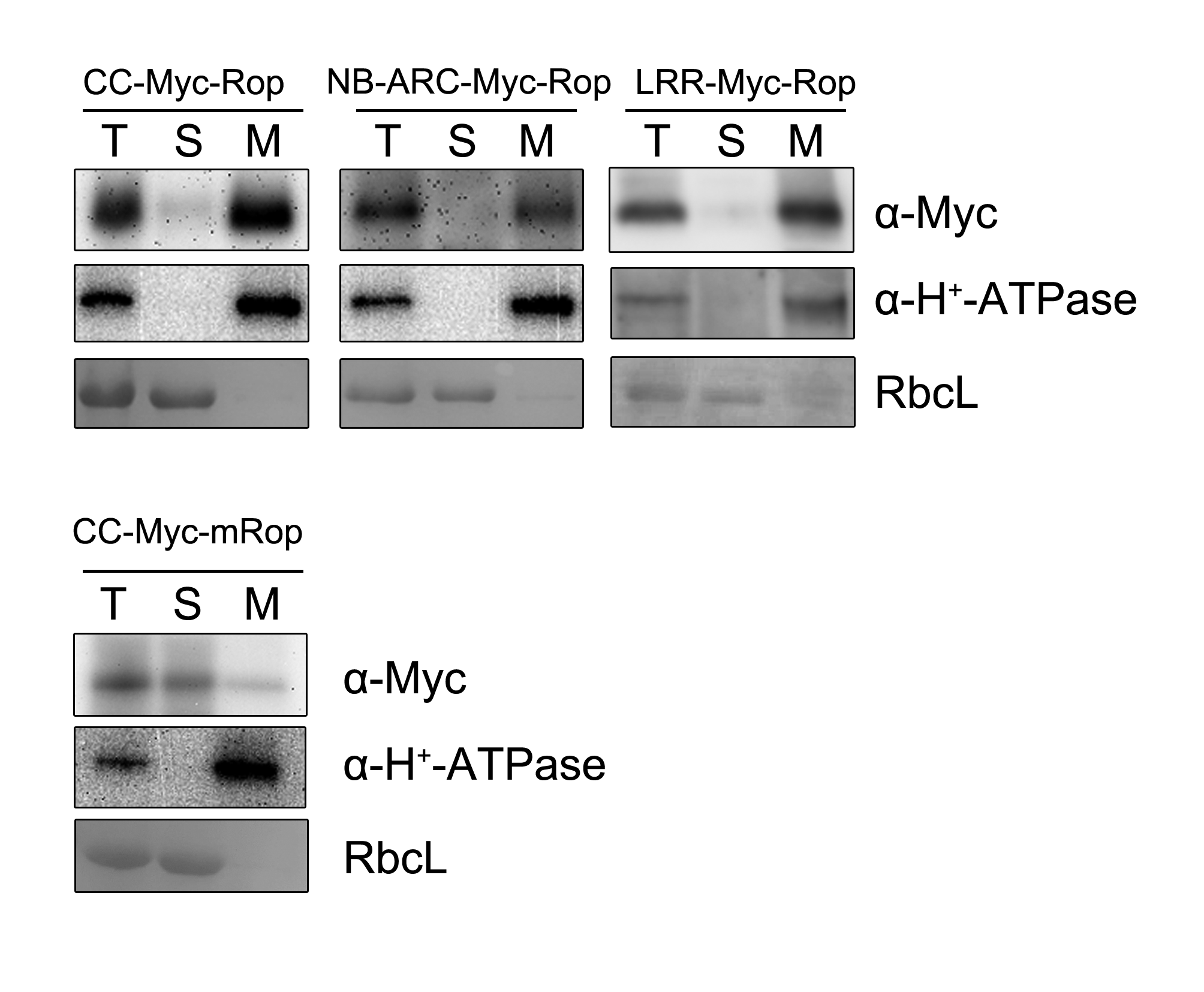

Supplement: S3 Fig — Cell lysates were separated to the soluble and microsomal membrane fractionations. T, total protein; S, soluble fraction; M, microsomal membrane fraction. H+-ATPase is the PM marker detected by IB, and RbcL is the soluble protein marker detected by Ponceau S. (TIF) [file ppat.1008475.s004.tif]

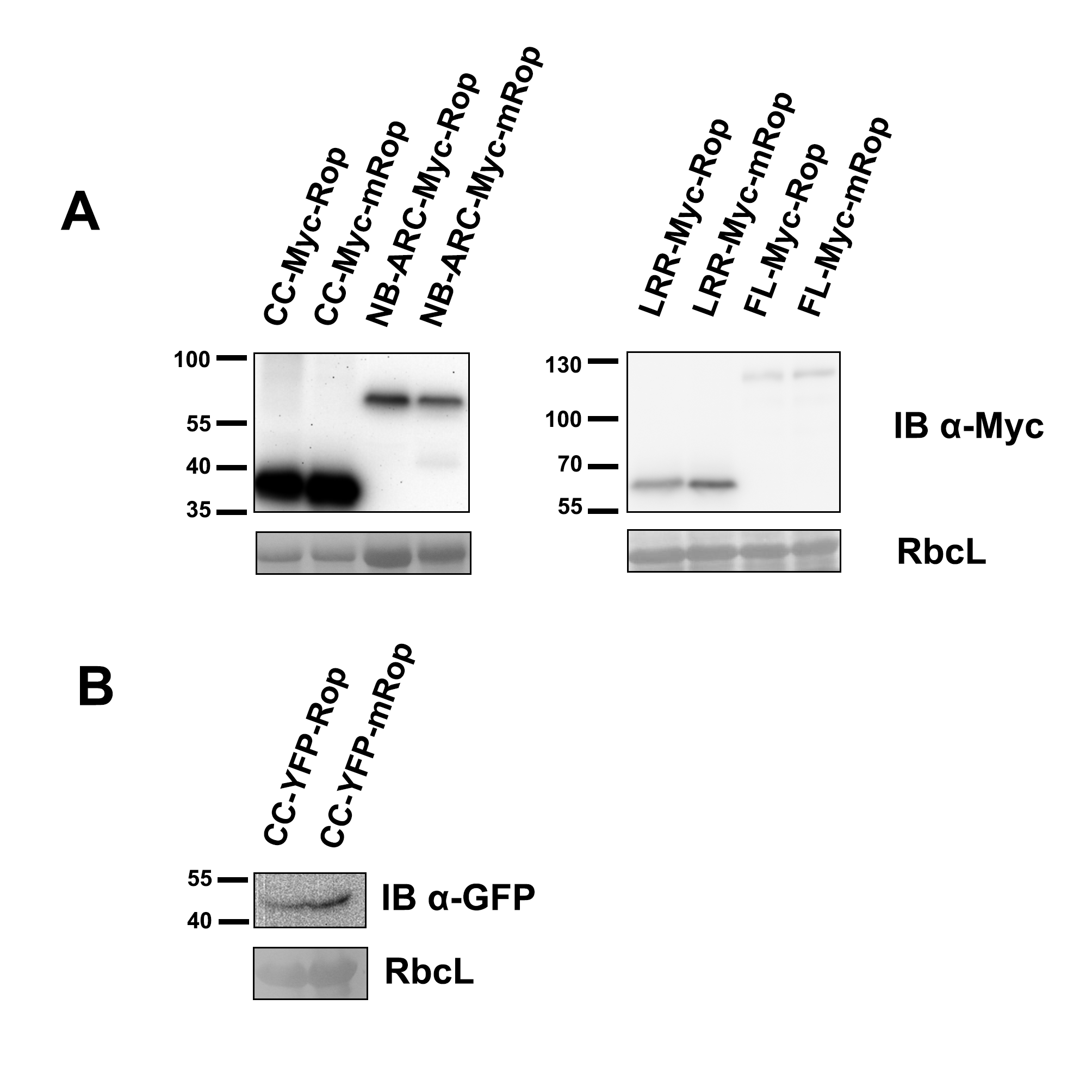

Supplement: S4 Fig — (A) Fusion proteins were probed with anti-Myc antibody. (B) Fusion proteins were probed with anti-GFP antibody. (TIF) [file ppat.1008475.s005.tif]

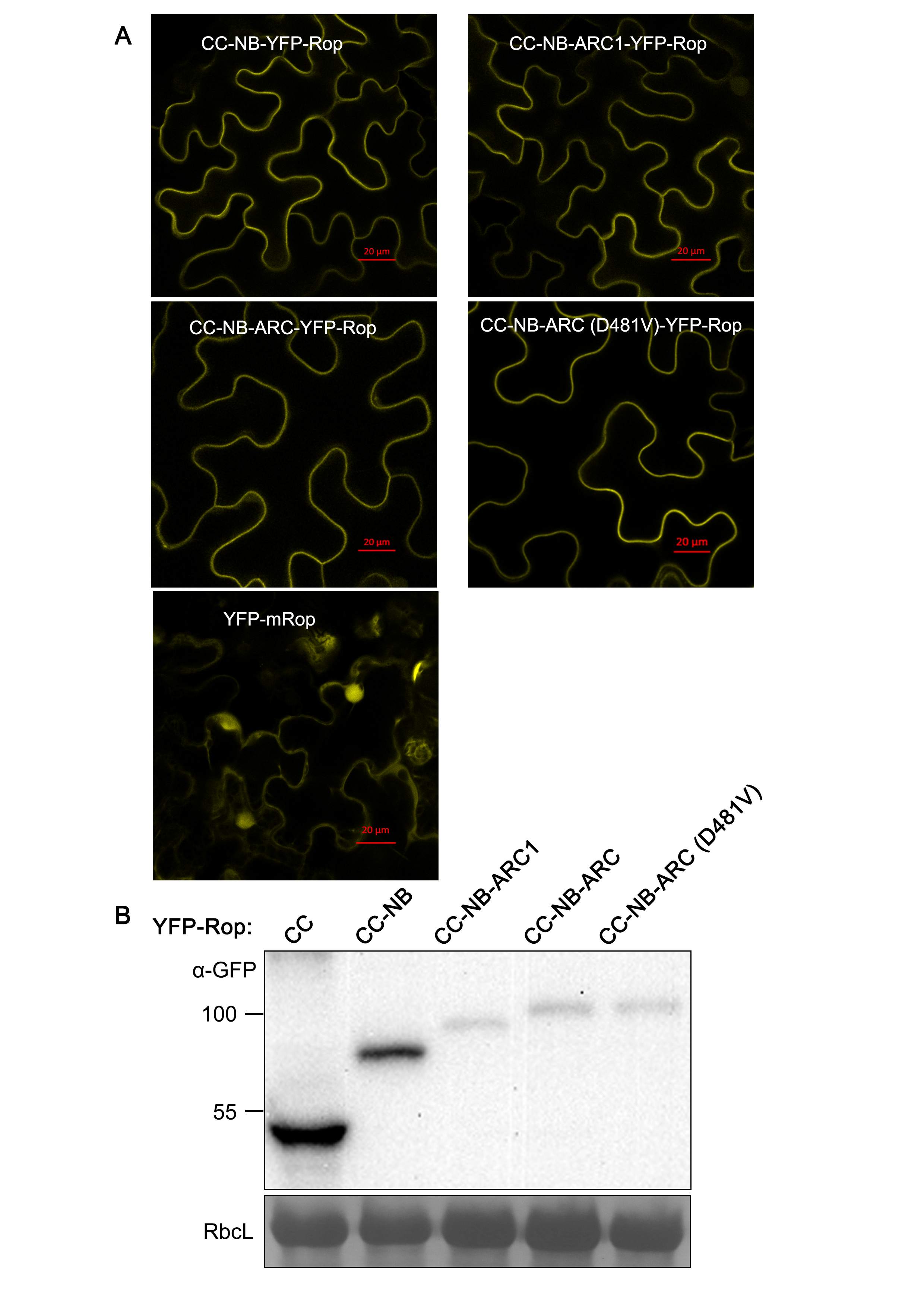

Supplement: S5 Fig — (A) Confocal images showed YFP-Rop successfully tethered fusion proteins to the PM. (B) Protein levels were detected by anti-GFP antibody. (TIF) [file ppat.1008475.s006.tif]

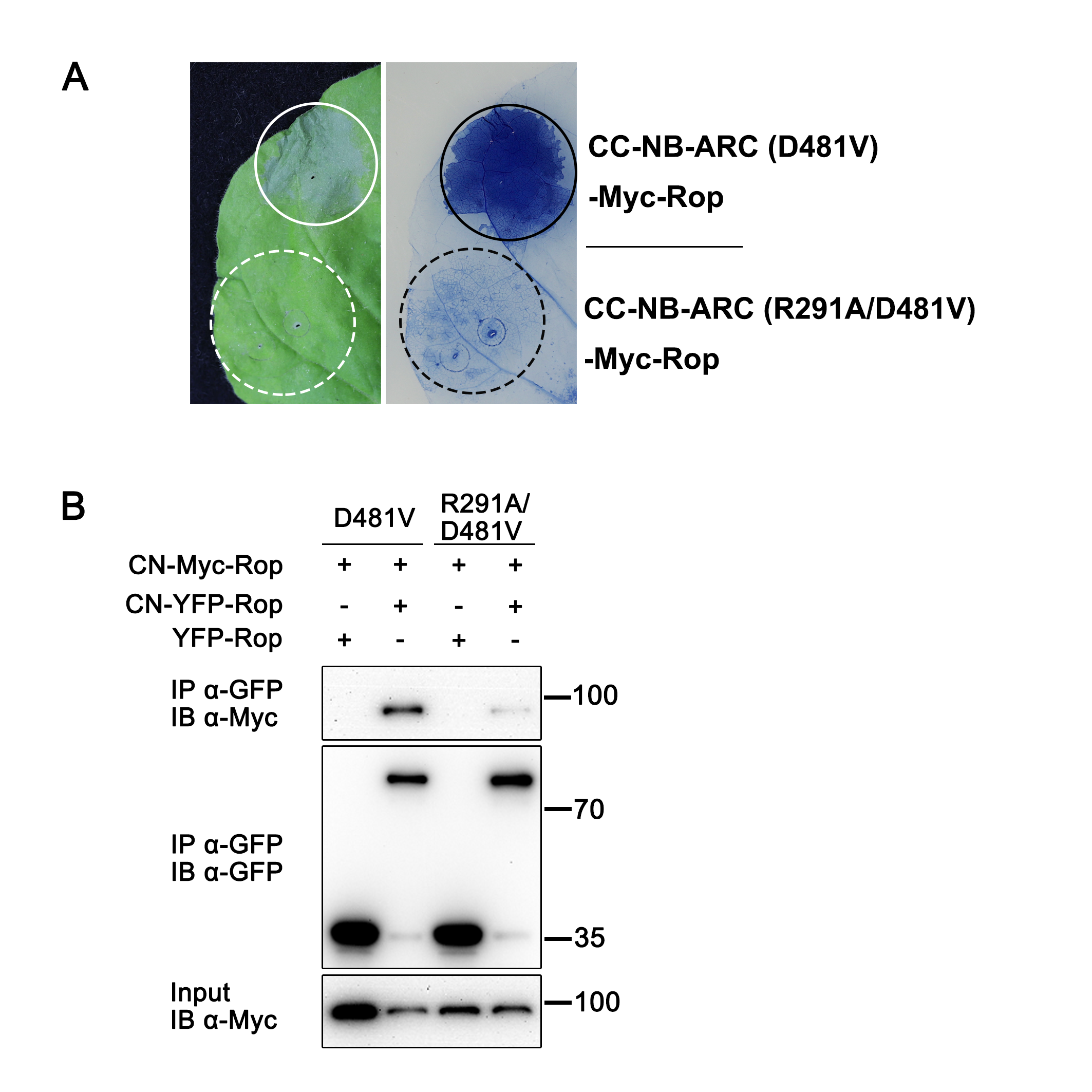

Supplement: S6 Fig — (A) R291A mutation inhibited cell death mediated by CC-NB-ARC (D481V)-Myc-Rop. Pictures were taken at 3 dpi. (B) R291A disrupted the self-association of CC-NB-ARC (D481V)-Rop. All samples were subjected to IP with anti-GFP beads. (TIF) [file ppat.1008475.s007.tif]

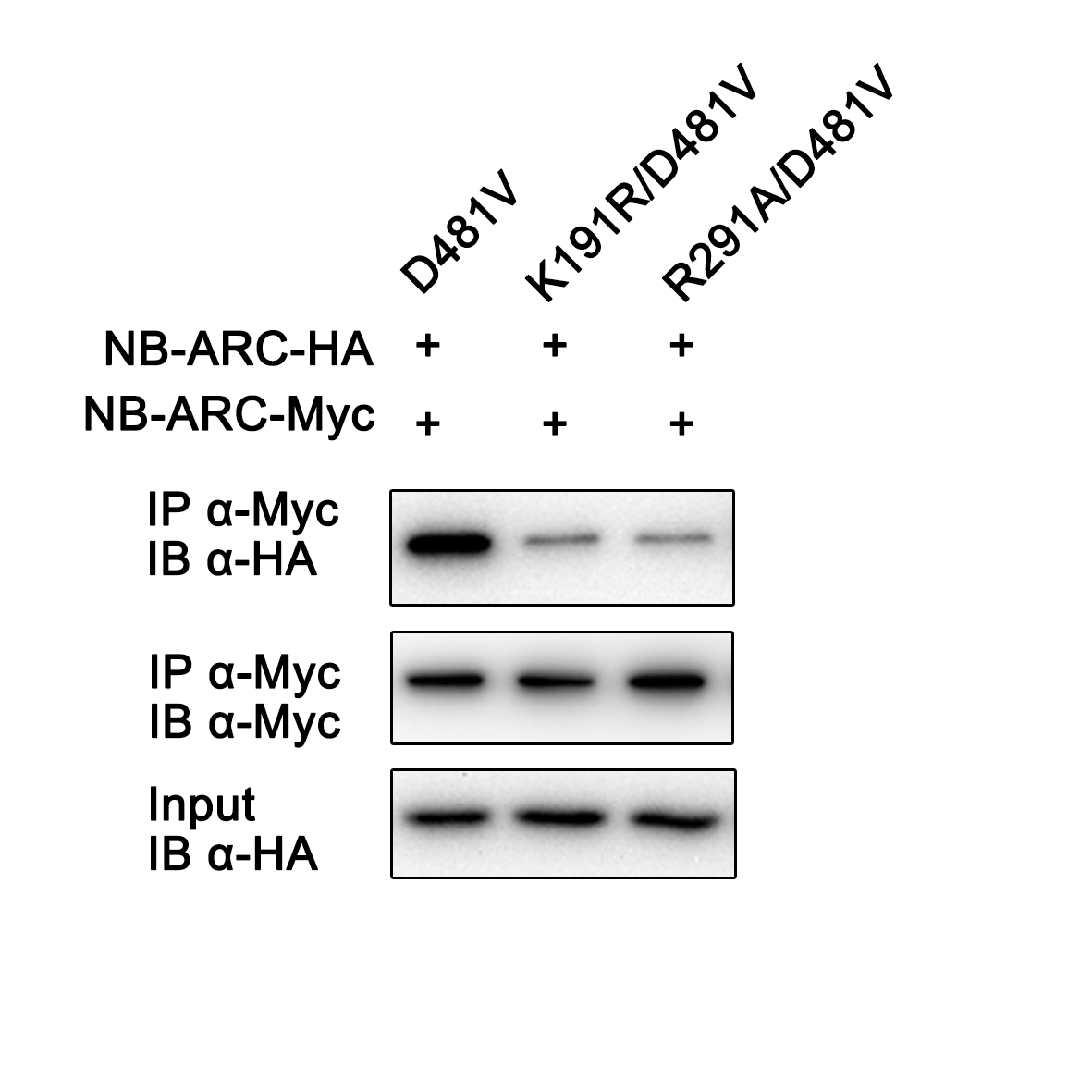

Supplement: S7 Fig — Co-IP assay showed that both K191R and R291A disrupted the self-association of NB-ARC (D481V). All samples were subjected to IP with anti-Myc beads. (TIF) [file ppat.1008475.s008.tif]

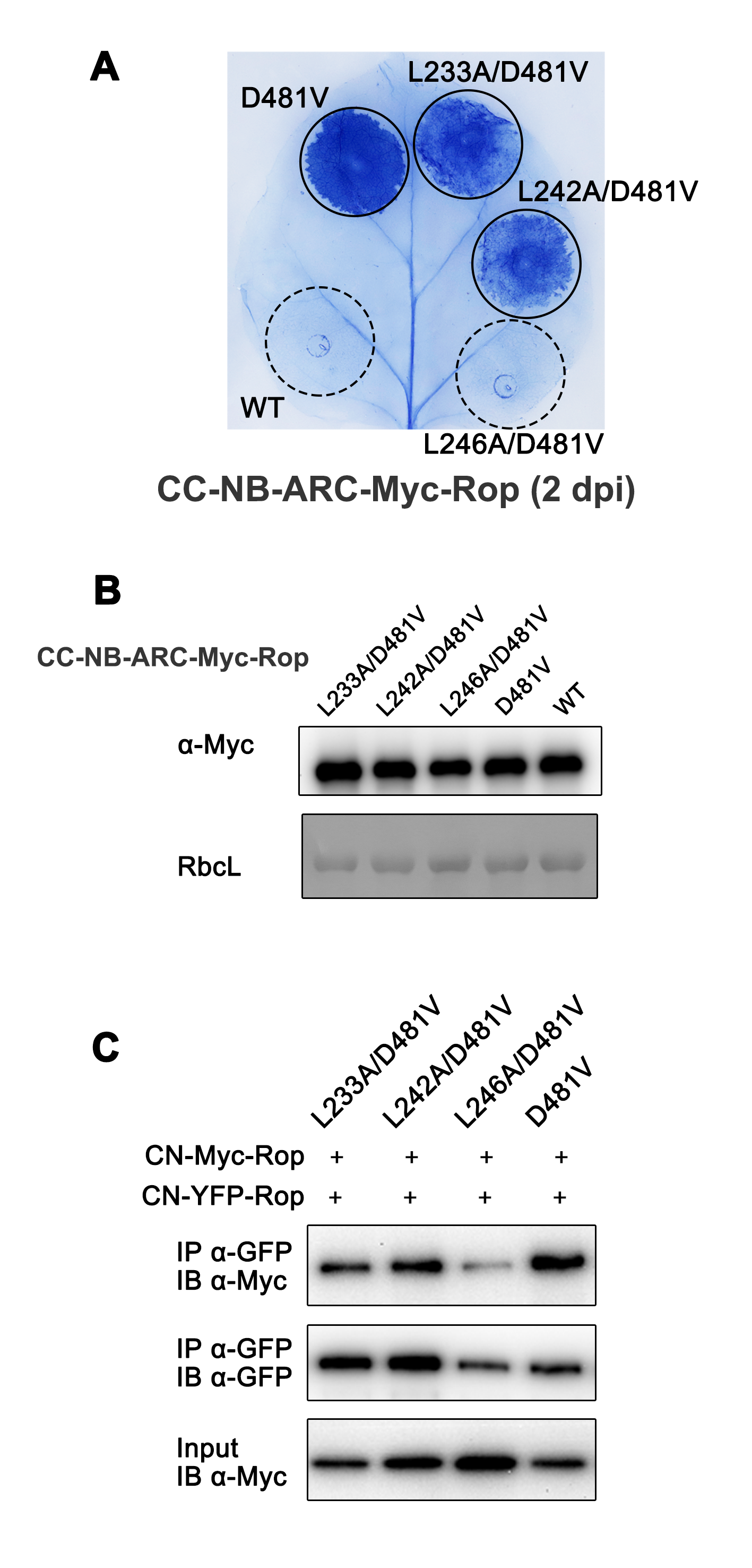

Supplement: S8 Fig — (A) Trypan blue staining showed that L233A, L242A and L246A disturbed the cell death phenotype induced by CC-NB-ARC (D481V)-Myc-Rop. (B) The expression of CC-NB-ARC-Myc-Rop and its mutants were detected by anti-Myc antibody. RbcL was stained by Ponceau S as loading control. (C) Co-IP assay showed that L233A, L242A and L246A disturbed the self-association of CC-NB-ARC (D481V)-Rop. All samples were subjected to IP with anti-GFP beads. (TIF) [file ppat.1008475.s009.tif]
